# MLEP: an R package for exploring the maximum likelihood estimates of penetrance parameters

**DOI:** 10.1186/1756-0500-5-465

**Published:** 2012-08-28

**Authors:** Yuki Sugaya

**Affiliations:** 1 School of Fundamental Science and Technology, Keio University, Yokohama, Japan

**Keywords:** Penetrance, Maximum likelihood estimate, Linkage analysis, Polynomial evaluation

## Abstract

**Background:**

Linkage analysis is a useful tool for detecting genetic variants that regulate a trait of interest, especially genes associated with a given disease. Although penetrance parameters play an important role in determining gene location, they are assigned arbitrary values according to the researcher’s intuition or as estimated by the maximum likelihood principle. Several methods exist by which to evaluate the maximum likelihood estimates of penetrance, although not all of these are supported by software packages and some are biased by marker genotype information, even when disease development is due solely to the genotype of a single allele.

**Findings:**

Programs for exploring the maximum likelihood estimates of penetrance parameters were developed using the R statistical programming language supplemented by external C functions. The software returns a vector of polynomial coefficients of penetrance parameters, representing the likelihood of pedigree data. From the likelihood polynomial supplied by the proposed method, the likelihood value and its gradient can be precisely computed. To reduce the effect of the supplied dataset on the likelihood function, feasible parameter constraints can be introduced into maximum likelihood estimates, thus enabling flexible exploration of the penetrance estimates. An auxiliary program generates a perspective plot allowing visual validation of the model’s convergence. The functions are collectively available as the MLEP R package.

**Conclusions:**

Linkage analysis using penetrance parameters estimated by the MLEP package enables feasible localization of a disease locus. This is shown through a simulation study and by demonstrating how the package is used to explore maximum likelihood estimates. Although the input dataset tends to bias the likelihood estimates, the method yields accurate results superior to the analysis using intuitive penetrance values for disease with low allele frequencies. MLEP is part of the Comprehensive R Archive Network and is freely available at http://cran.r-project.org/web/packages/MLEP/index.html.

## Findings

### Background

Linkage analysis remains a useful tool for detecting genetic variants that regulate a trait of interest, especially genes associated with a given disease. The likelihood of pedigree data plays an important role in this analysis; however, the entire likelihood function embodies functions of recombination fractions, penetrance parameters, and disease and marker allele frequencies. Such complexity can be reduced by varying only the recombination fraction, assigning fixed values to the other parameters. This allows the multivariate function to be expressed in terms of a single variable (the recombination fraction). The ratio of the univariate likelihood, the so-called LOD score [1], is then computed to map disease loci, rather than maximizing the likelihood itself. For a small recombination fraction, a high LOD score (greater than 3) implies that the disease locus is located near the markers employed in the analysis. Assuming the conditional parameters to achieve such high LOD scores depends largely on the researcher’s intuition.

Linkage analysis is hindered by the lack of useful tools and programs for the parameter estimation, although several methods for maximum likelihood estimate (MLE) of penetrance have been proposed. Two types of penetrance estimation are described in the literature. The first is based on pedigree data with marker genotype information; for which the likelihood is *p*_***θ***_(*a*_*V*_,*m*_*V*_), where *a*_*V *_and *m*_*V *_represent observations of affected status and marker genotypes respectively, for a set of pedigree members *V*. The parameter vector ***θ ***contains penetrance parameters, disease allele frequency, and recombination fraction, so that maximum likelihood estimates are obtained for all parameters simultaneously. The evaluated maximum likelihood estimate of penetrance parameters is therefore affected by the estimates of both recombination fraction and disease allele frequency. Because penetrance and marker genotype observations are independent (unless the marker and disease loci are extremely close), the method is not suitable for penetrance estimation in which a single disease allele determines whether the disease will manifest. This method has been shown in GENEHUNTER-MODSCORE [2-5] in which the ratio of the likelihood, or mod score function [6], can be maximized in practice. The second approach considers the likelihood of affected status; that is, the likelihood is expressed as $p_{\tilde{\theta }}(a_{V})$. Maximized likelihood is a function of the penetrance parameters alone, $\tilde{\theta }$, for a particular case. Wang et al. [7] formalized the likelihood using Bayes theorem and proposed a method for estimating the penetrance parameter, but which functions only when a carrier of a disease allele develops the disease. The estimate was applied to a dominant disease; therefore, it is not applicable to diseases which manifest via other modes of inheritance. Swartz et al. [8] developed three different kinds of estimators and compared their efficiencies by a ratio of asymptotic variance of one estimator to another. The efficiency was computed and illustrated by a perspective plot using the program Maple [9,10], but these methods are limited to sib pairs. The methods have not been packaged as freely available software, hence are not immediately applicable to wider data analysis. A known problem with penetrance estimation methods is that their estimates depend largely on the collected pedigrees. However, these second approaches ensure robust estimates by using multiple sets of previously-recorded pedigree data for the same disease to estimate parameters, which are available from the literature; the marker genotype information is not available.

Our proposed method belongs to the second class of penetrance estimation. The likelihood $p_{\tilde{\theta }}(a_{V})$ can be explicitly expressed as a polynomial of penetrance parameters, and the evaluation measure is a vector of likelihood polynomial coefficients. More precisely, the coefficient vectors for each pedigree are evaluated by their independence property. Once the likelihood vectors have been computed, the problem reduces to one of optimization. The maximum likelihood estimates of penetrance parameters are then readily obtained by maximizing the likelihood using standard statistical software. We have developed programs to explore the maximum likelihood estimates of penetrance parameters using R statistical programming language [11] supplemented with external C functions. The main function of the package evaluates the list of likelihood coefficient vectors. The software enables flexible exploration of the penetrance estimates by calculating the likelihood value and its gradient precisely, and passing them to an optimization function. The exploration is rendered more powerful if feasible parameter constraints can be incorporated in the maximum likelihood evaluation. Another advantage of our method is that it provides visual validation of convergence, in the form of perspective plots of the likelihood surface. All of the functions are available in the MLEP R package. Although the MLEP estimates are biased by the collected disease pedigree data, we show that they can more accurately identify a disease locus than can analysis based on intuitive penetrance values provided that both true and assumed disease allele frequency are small. The MLEP package is part of the Comprehensive R Archive Network (CRAN) and is freely downloadable from http://cran.r-project.org/web/packages/MLEP/index.html.

### Methods

First we introduce penetrance parameters as conditional probabilities given the genotype of the disease locus, such that *α *=* P*(*affected*|*A*/*A*), *β *=* P*(*affected*|*A*/*a*), and *γ *=* P*(*affected*|*a*/*a*), where *A* and *a* represent the disease and normal alleles respectively, and the genotype of the disease locus is expressed as two alleles separated by a slash. For simplicity, we suppose that a single locus contributes to disease development and that the disease allele frequency is known a priori. Using the above notations, the likelihood function for a pedigree is explicitly expressed as a polynomial of the parameters as follows:

$$L(\alpha ,\beta ,\gamma )=\underset{i,j,k}{\sum }c_{\text{ijk}}\alpha^{i}\beta^{j}\gamma^{k},$$

where *i*, *j*, and *k* run over from 0 to *N* subject to the constraint *max*(*i* + *j* + *k*) =* N *and *c*_*ijk*_ is a polynomial coefficient. Note that *N*, the number of individuals whose disease status (affected or unaffected) is known and the parameter constraint 0 ≤* γ *≤* β *≤* α *≤ 1 is reasonable. An individual of *A*/*a*genotype (where *A* is a dominant disease allele) has equal or higher chance of developing disease than an individual of *a*/*a* genotype, while the *A*/*A *genotype incurs the highest risk of disease development. Details of the likelihood evaluation algorithm are provided as Additional file 1. The polynomial form of a likelihood evaluation for the recombination fraction has been previously proposed and its usefulness has been demonstrated [12]. Here we present a modified form of the evaluation. Because the coefficient *c*_*ijk*_ is inherited through the generations from founder to descendant, the likelihood coefficients of penetrance parameters can similarly be evaluated.

### Evaluation of maximum likelihood estimate by MLEP

Here we use the MLEP package to explore the maximum likelihood estimate of penetrance parameters through a simulation study.

#### Data Simulation

We used the SLINK package [13,14] to generate simulation pedigree data. The pedigree structure followed that of Kamatani et al. [15] and the same phenotypes of the top two founders were used in the simulation, while the others were generated. All marker genotypes were also simulated by the program, assuming five marker alleles with equal frequencies. Allele frequency for the disease allele was assumed to be 0.0001. Three penetrance parameters (*α*,*β*,*γ*), with values 0.95, 0.7 and 0, were assigned, and the recombination frequency was assumed to be 0. By repeating the simulation under these conditions, 50 pedigree datasets were generated. All of the diseased individuals for each pedigree are assumed to have acquired their disease from a single locus.

#### Evaluation of likelihood polynomial

The main function of the MLEP package, mlep, evaluates the list of likelihood coefficient vectors of penetrance parameters. This function accepts a pedigree matrix consisting of the first six columns in a pedfile (the sixth column supplies affected status information), and returns a list of likelihood coefficients. To conserve computational memory, powers of penetrance parameters, *i*, *j*, and *k*, for a coefficient *c*_*ijk*_ are converted into a single value, *i* + *j *×* max*_*power* + *k *×* max*_*power*^2^; the converted values are then combined into a vector and assigned to the evaluated coefficients vector as a “*powers*” attribute, where “*max*_*power*" =* N* + 1. An additional attribute, “*max*_*power*”, maps the converted value to the original powers. After installing the MLEP package from the CRAN website and importing the simulation pedigree data into R as pedigree object, the package can be loaded and the mlep function can be executed by assuming the disease allele frequency to be 0.0001 as follows:

> library(MLEP)

> polynomial = mlep(pedigree, 0.0001)

#### Evaluation of maximum likelihood estimate

Once the likelihood polynomial has been evaluated by the mlep function, maximum likelihood estimates may be computed using any optimization program. Derivatives (and the Hessian) of the function are readily computed from the evaluated likelihood coefficient, hence the method presents as a powerful tool for seeking maximum likelihood. To this end, the functions fr and grr are provided for evaluation of the log likelihood value and its gradient. The likelihood polynomial is maximized by the pre-included R function constrOptim, which minimizes or maximizes a function subject to linear inequality constraints using an adaptive barrier algorithm [16]. Assuming the initial values of the parameters to be 0.9, 0.8, and 0.1, the constrOptim function can be executed to maximize the likelihood polynomial function, subject to 0 ≤* γ *≤* β *≤* α *≤ 1 as follows:

> constrOptim(c(0.9,0.8,0.1), fr, grr,

ui=rbind(c(1,0,0),c(0,1,0),c(0,0,1),

c(-1,0,0),c(0,-1,0),c(0,0,-1),

c(1,-1,0),c(0,1,-1)),

ci=c(rep(0,3), rep(-1,3), rep(0,2)),

poly=polynomial, control=list

(fnscale=-1), mu=0.01)

**Figure 1:**
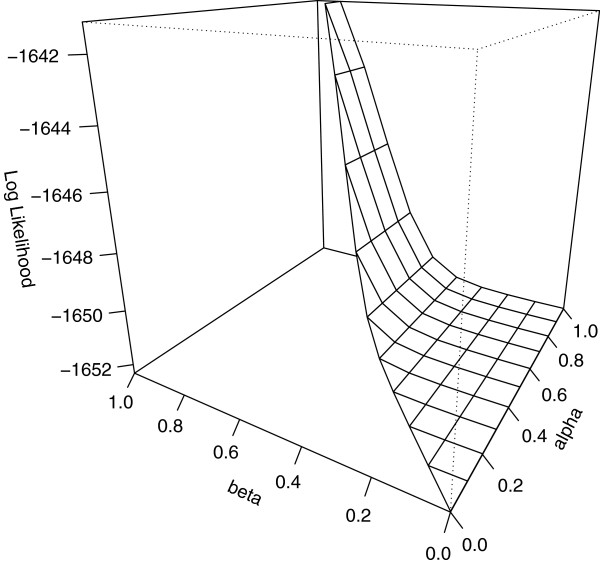
**Perspective plot of the log likelihood surface of the simulation pedigree data with *γ * = 0.188.** The simulated pedigree dataset is generated assuming penetrance values 0.95, 0.7, and 0, and disease allele frequency 0.0001. The likelihood of the simulated pedigree data is evaluated with frequency assigned to 0.0001, and the penetrances are estimated by the MLEP package. Fixing *γ *at its estimate 0.188, the log likelihood surface is drawn on a limited region reflecting the parameter constraint *α *≥* β*. The maximum appears near those of the other two maximum likelihood estimates (*α*,*β*) = (0.970,0.943).

By executing the above command, the maximum likelihood estimates were obtained as 0.970, 0.943, and 0.188 with log likelihood value -1640.339. It is important to check whether the iteration converges to the global maximum. To this end, we use the PerspPenetrance function, which draws a perspective plot of the log likelihood surface fixed on one of the parameters. A plot of the evaluated likelihood for fixed *γ *= 0.188 is shown in Figure 1. The plot is generated from the following command:

> PerspPenetrance(polynomial, "gamma",

0.188, theta=-60, phi=20)

Although the maximum is found near those of the other two maximum likelihood estimates, *α* and *β*, evaluated in this study, the estimates appear to be biased. This bias is not inherent in the model, but is introduced by the input data. The moderate probability of disease development for individuals of *A*/*a* disease genotype, 0.7, has likely produced the bias. If strong evidence exists that the probability of disease development for individuals of *a*/*a* genotype is low (,that is, phenocopy rate is low), a second command can be executed by changing one of the linear inequality constraints and the initial value of *γ *to 0 ≤* γ *≤ 0.01 and 0.001 as follows:

> constrOptim(c(0.9,0.8,0.001), fr, grr,

ui=rbind(c(1,0,0),c(0,1,0),c(0,0,1),

c(-1,0,0),c(0,-1,0),c(0,0,-1),

c(1,-1,0),c(0,1,-1)),

ci=c(rep(0,3), rep(-1,2), -0.01,

rep(0,2)), poly=polynomial,

control=list(fnscale=-1), mu=0.01)

**Figure 2:**
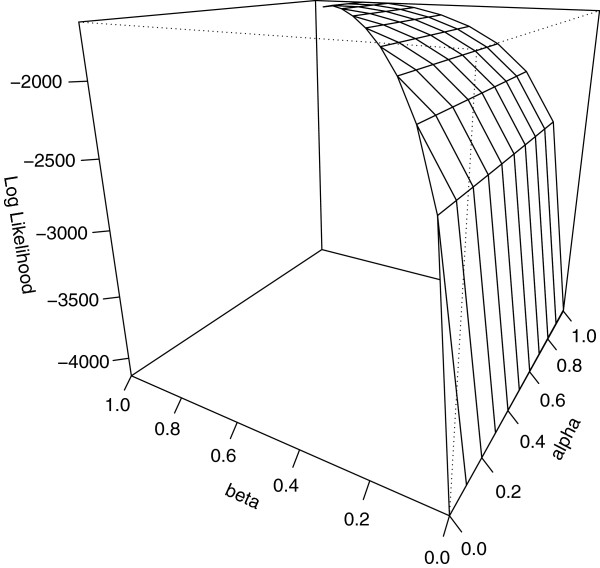
**Perspective plot of the log likelihood surface of the simulation pedigree data with *γ *= 0.003.** The same likelihood polynomial as that of Figure 1 is plotted with the *γ*penetrance estimate fixed at 0.003. The penetrance estimates are evaluated employing a parameter constraint 0 ≤* γ *≤ 0.01. The maximum appears near those of the other two estimates (*α*,*β*) = (0.875,0.759).

Following this adjustment, the obtained maximum likelihood estimates were 0.875, 0.759, and 0.003 with log likelihood value -1644.840 less that of global maximum. The perspective plot for fixed *γ *= 0.003 is shown in Figure 2 and the maximum is found near those of the other two estimates although the surface is flat around the maximum.

#### Power Comparison

To assess linkage analysis efficacy for different penetrance values, the expected LOD score was evaluated by the SLINK package given the following four penetrance models: true penetrance model (0.950, 0.700, and 0.000), dominant model (0.999, 0.999, and 0.000), MLE with the *γ *constraint model (0.875, 0.759, and 0.003), and unrestrained MLE model (0.970, 0.943, and 0.188). The results are summarized in Table 1. In the true penetrance model, the highest LOD score (exceeding 3) is correctly obtained at *θ *= 0. The dominant model, which is often used for simplicity in practical analysis, yields the highest LOD score at *θ *= 0.1. Therefore, researchers may conclude that the disease locus exists relatively close, but not extremely close to, the employed marker. This example highlights the potential of intuitive approaches to yield an incorrect result. Analysis via MLE constrained by the *γ *parameter yields results that match the true model, but unconstrained MLE leads to the uncertain conclusion that the disease locus may not exist near the employed marker, since the maximum LOD score at *θ *= 0.05 in this case is less than 3.

**Table 1:** Summary of LOD scores for the four parameter models

Model	**Recombination fraction(*θ*)**									
	0	0.05	0.1	0.15	0.2	0.25	0.3	0.35	0.4	0.45
True	4.071	3.831	3.535	3.201	2.835	2.438	2.011	1.553	1.066	0.549
Dominant	-0.158	3.296	3.394	3.240	2.965	2.609	2.189	1.714	1.189	0.617
MLE (with *γ* constraint)	3.992	3.797	3.520	3.198	2.838	2.444	2.017	1.559	1.069	0.550
MLE (unconstrained)	0.781	0.792	0.710	0.603	0.487	0.370	0.260	0.167	0.095	0.041

### Effect of misspecifying disease allele frequency

In the above simulation study, we assigned the disease allele frequency to the value used to simulate the pedigree data (referred to as the “true” value in this paper) to evaluate the likelihood polynomial, but the extent to which this value matches reality is unknown. To evaluate the effect of misspecification of disease allele frequency on maximum likelihood estimates of penetrance parameters and on the subsequent linkage analysis, we altered both true and assumed disease allele frequencies in the following simulation study.

#### Data Simulation

The pedigree structure, number of marker alleles, marker allele frequencies, penetrance parameters, and recombination fractions were identical to those used in the previous simulation, while disease allele frequency was altered to 0.0001, 0.001, 0.01, 0.1, or 0.2. 50 pedigree datasets were generated for each disease allele frequency. For the simulated pedigree datasets, disease allele frequency was then assumed to be 0.0001, 0.001, 0.01, 0.1, 0.25, or 0.5, and the likelihood polynomials were evaluated in each case.

#### Maximum likelihood estimate

For each of the evaluated likelihood polynomials, we also evaluated their maximum likelihood estimates subject to the constraint 0 ≤* γ *≤ 0.01. The unconstrained estimates for disease allele frequency 0.0001 displayed similar bias to the assumed frequency in the previous simulation study. Hence we employed the same parameter constraints as previously applied for all cases in the present simulation, although the constraint exerted little effect on the likelihood estimates at disease allele frequencies greater than 0.0001. The evaluated maximum likelihood estimates, mean absolute bias, and mean squared error (MSE) of the estimates are summarized in Table 2. Note that *γ* is restricted to small values. When the true frequency is 0.0001, 0.001, or 0.01, and the likelihood is also evaluated at one of these frequencies, we find that *α* is underestimated and *β *is overestimated. If the likelihood is evaluated at frequencies exceeding 0.01, the estimates become unstable as both the mean bias and MSE become large. Assuming the true frequency to be 0.1 or 0.2 but evaluating the likelihood at frequencies less than 0.1 results in overestimation of both *α* and *β*. If the likelihood is evaluated at frequency 0.1, the estimates remain biased, but likelihood estimates evaluated at higher allele frequencies become unstable. Perspective plots of the resulting log likelihood surfaces at fixed *γ* are provided as Additional file 2. Although the estimates are biased or unstable, the maxima are correctly located near those of the other two estimates for all cases studied.

**Table 2:** Summary of maximum likelihood estimates of penetrance parameters


**True value**	**0.0001**					
**Assumed value**	**0.0001**	**0.001**	**0.01**	**0.1**	**0.25**	**0.5**
MLE	(0.832,0.759,0.003)	(0.766,0.757,0.002)	(0.749,0.749,0.001)	(0.690,0.688,0.000)	(0.989,0.159,0.000)	(0.602,0.002,0.000)
Bias	(0.118,0.112,0.002)	(0.124,0.112,0.002)	(0.145,0.110,0.002)	(0.188,0.143,0.001)	(0.204,0.379,0.001)	(0.330,0.628,0.001)
MSE	(0.020,0.020,0.000)	(0.023,0.020,0.000)	(0.035,0.020,0.000)	(0.064,0.030,0.000)	(0.082,0.183,0.000)	(0.142,0.415,0.000)
**True value**	**0.001**					
**Assumed value**	**0.0001**	**0.001**	**0.01**	**0.1**	**0.25**	**0.5**
MLE	(0.832,0.759,0.003)	(0.766,0.757,0.002)	(0.749,0.748,0.001)	(0.617,0.614,0.000)	(0.916,0.208,0.000)	(0.592,0.003,0.000)
Bias	(0.118,0.112,0.002)	(0.124,0.112,0.002)	(0.145,0.111,0.002)	(0.215,0.135,0.001)	(0.213,0.380,0.001)	(0.360,0.656,0.000)
MSE	(0.020,0.020,0.000)	(0.023,0.020,0.000)	(0.035,0.020,0.000)	(0.076,0.026,0.000)	(0.085,0.180,0.000)	(0.149,0.441,0.000)
**True value**	**0.01**					
**Assumed value**	**0.0001**	**0.001**	**0.01**	**0.1**	**0.25**	**0.5**
MLE	(0.772,0.767,0.010)	(0.766,0.765,0.008)	(0.753,0.753,0.002)	(0.632,0.612,0.000)	(0.880,0.279,0.000)	(0.586,0.000,0.000)
Bias	(0.111,0.110,0.003)	(0.114,0.110,0.003)	(0.132,0.109,0.003)	(0.191,0.127,0.001)	(0.197,0.365,0.001)	(0.356,0.648,0.000)
MSE	(0.017,0.020,0.000)	(0.019,0.020,0.000)	(0.031,0.019,0.000)	(0.063,0.024,0.000)	(0.075,0.170,0.000)	(0.145,0.430,0.000)
**True value**	**0.01**					
**Assumed value**	**0.0001**	**0.001**	**0.01**	**0.1**	**0.25**	**0.5**
MLE	(0.972,0.802,0.010)	(0.962,0.804,0.010)	(0.925,0.799,0.010)	(0.776,0.680,0.000)	(0.764,0.440,0.000)	(0.591,0.017,0.000)
Bias	(0.083,0.116,0.007)	(0.095,0.118,0.007)	(0.106,0.116,0.006)	(0.125,0.111,0.002)	(0.181,0.269,0.002)	(0.272,0.588,0.001)
MSE	(0.011,0.020,0.000)	(0.013,0.021,0.000)	(0.018,0.021,0.000)	(0.033,0.020,0.000)	(0.056,0.102,0.000)	(0.099,0.373,0.000)
**True value**	**0.2**					
**Assumed value**	**0.0001**	**0.001**	**0.01**	**0.1**	**0.25**	**0.5**
MLE	(0.995,0.765,0.010)	(0.998,0.777,0.010)	(0.964,0.778,0.010)	(0.697,0.647,0.005)	(0.719,0.381,0.000)	(0.631,0.074,0.000)
Bias	(0.104,0.129,0.009)	(0.104,0.130,0.009)	(0.108,0.139,0.008)	(0.142,0.132,0.005)	(0.203,0.220,0.003)	(0.308,0.579,0.002)
MSE	(0.018,0.026,0.000)	(0.019,0.026,0.000)	(0.023,0.028,0.000)	(0.043,0.030,0.000)	(0.070,0.071,0.000)	(0.124,0.351,0.000)

#### Power of detecting linkage

Based on the evaluated maximum likelihood estimates, the expected LOD scores for the following three penetrance models were evaluated by the SLINK packages; true penetrance model (0.950, 0.700, and 0.000), dominant model (0.999, 0.999, and 0.000), and MLE model (the estimates are listed in Table 2). The results are shown in Table 3. The true and MLE models yield maximum LOD scores greater than 3 at *θ *= 0 when both true and assumed disease allele frequencies are 0.0001, 0.001, or 0.01, or when the true frequency is one of these values and the assumed frequency is 0.1. These models performed with similar correctness at true frequency 0.1 and assumed frequency 0.01 or 0.1. The dominant model, on the other hand, yielded poor or misplaced LOD scores for all cases.

**Table 3:** Summary of LOD scores for the three penetrance models

**Disease allele frequency**		**Model**	**Recombination fraction(*θ*)**									
**True value**	**Assumed value**		**0**	**0.05**	**0.1**	**0.15**	**0.2**	**0.25**	**0.3**	**0.35**	**0.4**	**0.45**
0.0001		True	4.071	3.831	3.535	3.201	2.835	2.438	2.011	1.553	1.066	0.549
	0.0001	MLE	3.992	3.797	3.520	3.198	2.838	2.444	2.017	1.559	1.069	0.550
		Dominant	-0.158	3.296	3.394	3.240	2.965	2.609	2.189	1.714	1.189	0.617
		True	4.065	3.826	3.530	3.196	2.830	2.434	2.007	1.550	1.063	0.547
	0.001	MLE	4.003	3.805	3.527	3.204	2.843	2.448	2.021	1.561	1.071	0.550
		Dominant	-0.188	3.265	3.364	3.211	2.939	2.586	2.170	1.698	1.176	0.609
		True	4.022	3.784	3.489	3.157	2.792	2.398	1.974	1.521	1.040	0.532
	0.01	MLE	3.984	3.780	3.500	3.175	2.815	2.421	1.995	1.538	1.051	0.538
		Dominant	-0.234	3.217	3.320	3.171	2.902	2.552	2.138	1.670	1.155	0.598
		True	3.702	3.474	3.191	2.872	2.524	2.150	1.750	1.328	0.887	0.437
	0.1	MLE	3.665	3.429	3.143	2.823	2.476	2.103	1.708	1.292	0.860	0.422
		Dominant	-0.425	3.040	3.146	3.002	2.738	2.397	1.997	1.549	1.059	0.534
		True	3.257	3.049	2.787	2.492	2.171	1.827	1.464	1.087	0.704	0.331
	0.25	MLE	1.524	1.407	1.270	1.121	0.964	0.800	0.634	0.467	0.303	0.146
		Dominant	-0.743	2.748	2.862	2.727	2.478	2.158	1.783	1.362	0.906	0.437
		True	2.549	2.384	2.165	1.917	1.646	1.359	1.061	0.760	0.469	0.205
	0.5	MLE	-0.009	0.204	0.292	0.321	0.315	0.284	0.238	0.182	0.121	0.059
		Dominant	-1.339	2.208	2.349	2.250	2.044	1.765	1.434	1.067	0.682	0.311
0.001		True	4.086	3.846	3.548	3.213	2.846	2.447	2.019	1.560	1.070	0.551
	0.0001	MLE	4.008	3.812	3.534	3.210	2.849	2.453	2.025	1.565	1.073	0.552
		Dominant	-0.140	3.312	3.409	3.253	2.977	2.619	2.198	1.721	1.193	0.619
		True	4.081	3.841	3.543	3.208	2.841	2.443	2.014	1.556	1.067	0.549
	0.001	MLE	4.018	3.820	3.541	3.216	2.854	2.458	2.028	1.567	1.075	0.553
		Dominant	-0.170	3.281	3.379	3.225	2.951	2.597	2.179	1.705	1.180	0.611
		True	4.038	3.799	3.502	3.169	2.803	2.407	1.981	1.527	1.044	0.534
	0.01	MLE	4.000	3.795	3.513	3.187	2.825	2.429	2.002	1.543	1.055	0.540
		Dominant	-0.216	3.234	3.335	3.185	2.915	2.563	2.147	1.677	1.160	0.601
		True	3.717	3.489	3.204	2.884	2.535	2.159	1.757	1.334	0.891	0.439
	0.1	MLE	3.602	3.338	3.040	2.717	2.372	2.006	1.621	1.221	0.809	0.394
		Dominant	-0.407	3.056	3.161	3.016	2.751	2.407	2.006	1.556	1.064	0.536
		True	3.272	3.063	2.800	2.503	2.181	1.836	1.471	1.093	0.708	0.332
	0.25	MLE	1.847	1.667	1.479	1.286	1.090	0.893	0.698	0.508	0.325	0.154
		Dominant	-0.725	2.765	2.877	2.741	2.490	2.168	1.792	1.369	0.911	0.439
		True	2.563	2.397	2.177	1.927	1.655	1.366	1.067	0.765	0.471	0.207
	0.5	MLE	0.048	0.234	0.310	0.332	0.321	0.288	0.240	0.183	0.121	0.060
		Dominant	-1.321	2.225	2.364	2.264	2.056	1.776	1.443	1.073	0.686	0.312
**0.01**		True	3.808	3.619	3.348	3.038	2.694	2.317	1.909	1.472	1.008	0.517
	0.0001	MLE	3.647	3.461	3.206	2.913	2.588	2.231	1.841	1.421	0.973	0.499
		Dominant	-0.374	3.002	3.150	3.037	2.794	2.466	2.072	1.621	1.122	0.580
		True	3.863	3.639	3.355	3.038	2.691	2.313	1.905	1.469	1.005	0.515
	0.001	MLE	3.730	3.532	3.267	2.964	2.628	2.262	1.864	1.437	0.983	0.503
		Dominant	-0.345	3.011	3.136	3.013	2.770	2.444	2.053	1.605	1.109	0.572
		True	3.881	3.637	3.344	3.018	2.664	2.284	1.876	1.442	0.983	0.501
	0.01	MLE	3.836	3.623	3.344	3.026	2.677	2.299	1.891	1.455	0.992	0.505
		Dominant	-0.329	3.019	3.133	2.998	2.746	2.417	2.025	1.579	1.089	0.562
		True	3.629	3.389	3.101	2.782	2.436	2.067	1.677	1.268	0.844	0.415
	0.1	MLE	3.514	3.243	2.944	2.622	2.281	1.922	1.548	1.162	0.767	0.374
		Dominant	-0.455	2.907	3.021	2.884	2.628	2.297	1.909	1.478	1.008	0.508
		True	3.224	3.002	2.734	2.437	2.115	1.775	1.418	1.050	0.679	0.319
	0.25	MLE	2.100	1.888	1.668	1.445	1.219	0.993	0.772	0.557	0.354	0.166
		Dominant	-0.737	2.651	2.774	2.647	2.405	2.093	1.728	1.320	0.879	0.426
		True	2.563	2.384	2.158	1.905	1.632	1.345	1.049	0.752	0.465	0.205
	0.5	MLE	-0.105	0.150	0.249	0.286	0.286	0.262	0.221	0.170	0.114	0.056
		Dominant	-1.289	2.160	2.311	2.219	2.018	1.746	1.422	1.062	0.684	0.315
0.1		True	2.571	3.140	2.984	2.742	2.445	2.105	1.730	1.326	0.900	0.457
	0.0001	MLE	2.645	2.809	2.691	2.494	2.245	1.955	1.632	1.276	0.886	0.460
		Dominant	-3.354	2.123	2.564	2.607	2.467	2.211	1.869	1.462	1.005	0.513
		True	2.965	3.191	3.011	2.753	2.447	2.103	1.727	1.323	0.897	0.455
	0.001	MLE	2.986	3.054	2.898	2.663	2.377	2.051	1.692	1.304	0.891	0.456
		Dominant	-2.980	2.115	2.538	2.580	2.442	2.189	1.850	1.446	0.991	0.504
		True	3.319	3.265	3.044	2.763	2.441	2.086	1.705	1.300	0.877	0.442
	0.01	MLE	3.223	3.185	2.991	2.731	2.423	2.077	1.701	1.299	0.876	0.441
		Dominant	-2.599	2.149	2.524	2.555	2.415	2.161	1.823	1.420	0.971	0.495
		True	3.388	3.185	2.916	2.609	2.274	1.915	1.538	1.149	0.754	0.366
	0.1	MLE	3.350	3.133	2.859	2.551	2.218	1.863	1.492	1.111	0.727	0.351
		Dominant	-2.344	2.159	2.479	2.473	2.314	2.051	1.713	1.320	0.894	0.446
		True	3.111	2.897	2.627	2.327	2.003	1.662	1.311	0.956	0.608	0.283
	0.25	MLE	2.471	2.224	1.967	1.702	1.434	1.165	0.900	0.644	0.403	0.186
		Dominant	-2.443	1.991	2.296	2.283	2.120	1.861	1.536	1.169	0.774	0.372
		True	2.561	2.370	2.128	1.859	1.573	1.276	0.978	0.688	0.419	0.184
	0.5	MLE	0.240	0.324	0.351	0.344	0.315	0.271	0.217	0.160	0.102	0.048
		Dominant	-2.804	1.584	1.892	1.894	1.751	1.522	1.244	0.929	0.596	0.275
0.2		True	1.245	2.462	2.452	2.312	2.096	1.827	1.517	1.174	0.804	0.412
	0.0001	MLE	1.471	2.049	2.086	2.006	1.852	1.641	1.383	1.086	0.754	0.392
		Dominant	-5.718	1.358	1.978	2.131	2.078	1.898	1.628	1.290	0.898	0.465
		True	1.891	2.542	2.488	2.325	2.099	1.826	1.514	1.171	0.801	0.410
	0.001	MLE	2.027	2.354	2.310	2.174	1.976	1.730	1.444	1.122	0.772	0.397
		Dominant	-5.102	1.341	1.948	2.100	2.048	1.869	1.602	1.268	0.883	0.458
		True	2.484	2.669	2.544	2.343	2.094	1.808	1.491	1.147	0.781	0.397
	0.01	MLE	2.435	2.557	2.448	2.263	2.030	1.757	1.451	1.116	0.759	0.385
		Dominant	-4.507	1.360	1.924	2.059	1.997	1.816	1.552	1.227	0.851	0.436
		True	2.763	2.662	2.461	2.218	1.944	1.647	1.332	1.002	0.663	0.323
	0.1	MLE	2.684	2.539	2.326	2.081	1.813	1.526	1.226	0.916	0.602	0.291
		Dominant	-4.074	1.352	1.847	1.951	1.876	1.694	1.435	1.117	0.756	0.379
		True	2.570	2.425	2.215	1.972	1.705	1.423	1.128	0.828	0.529	0.246
	0.25	MLE	1.948	1.755	1.551	1.341	1.128	0.916	0.707	0.506	0.317	0.146
		Dominant	-4.071	1.211	1.691	1.789	1.717	1.540	1.290	0.986	0.657	0.320
		True	2.101	1.964	1.775	1.560	1.328	1.085	0.837	0.591	0.360	0.159
	0.5	MLE	0.625	0.595	0.545	0.484	0.416	0.343	0.269	0.195	0.125	0.060
		Dominant	-4.299	0.868	1.353	1.466	1.413	1.260	1.039	0.781	0.509	0.239

These results indicate that genetic diseases with low allele frequencies (< 0.01) are suitable for analysis by linkage analysis; that is, the linkage between disease locus and marker locus can be correctly identified. At disease allele frequencies exceeding 0.1, the correct conclusion may not be reached because the peak of the significant LOD score (greater than 3) is obtained at different recombination fractions or because the linkage-detecting efficacy is insufficient (i.e., no LOD score higher than 3 is obtained) even when true penetrance parameters are employed. The MLEP package will almost certainly yield correct results for genetic diseases with small allele frequency, provided that the frequency is also assumed to be 0.1 or less. In this case, incorrectly specifying the disease allele frequency will have little effect on the results. When the true frequency is 0.1, assigning a small frequency (of order 0.01) to likelihood evaluations will again yield the correct result. In all other cases, the effect of allele frequency misspecification cannot be neglected. These results were validated by another simulation study, in which true penetrance parameters were assumed to be 0.990, 0.900, and 0.000. Maximum likelihood estimates, LOD scores for the three penetrance models, and perspective plots are provided as Additional files 3, 4, and 5, respectively.

### Discussion

We have developed the MLEP R package to explore maximum likelihood estimates of penetrance parameters. The polynomial form of the likelihood evaluation enables flexible exploration of penetrance estimates by evaluating both the likelihood value and its gradient precisely, and by introducing parameter constraints if these can be assumed. Introduction of a low phenocopy rate (*γ *< 0.01) especially improved the quality of the analysis result in the presented simulation study. Convergence may be verified visually by auxiliary perspective plots. The input pedigree datasets bias the evaluated maximum likelihood estimates; however, the likelihood surface may be flat around the maximum and the performance of the estimates at correctly identifying linkages is superior to that of intuitive penetrance values.

Currently, common linkage analysis employs dense marker data. The proposed method is applicable to such vast datasets because it treats only the likelihood that the disease status of a pedigree member is “affected”, and is independent of dataset size, which is derived from the marker genotypes. Penetrance parameters are estimated separately from the marker information, and linkage analysis employing the estimates is conducted as the next step. The method is applicable only to diseases with large effect size, that is, when a single disease locus greatly or wholly contributes to disease development. Diseases resulting from other mechanisms, such as multifactorial diseases, are not suitable for analysis by this approach. Disease allele frequency is another unknown important parameter in linkage analysis, but several previous studies have reported that misspecifying the disease allele frequency does not significantly influence to the detection of the linkage [6,17]. Our simulation study supports these results, provided that both true and assumed disease allele frequencies are small. It has also been mentioned that linkage analysis would be successful if a disease allele frequency is rare in the population [18]; therefore, assigning the frequency to a small value for such the disease produces a feasible result. Here, we have demonstrated that linkage analysis using maximum likelihood estimates by the MLEP package correctly localizes a disease locus, if the frequency of the disease allele is made small (< 0.1).

## Availability and requirements

**Project name:** MLEP

**Project home page:**http://cran.r-project.org/web/packages/MLEP/index.html

http://www.stat.math.keio.ac.jp/∼sugaya/PIA/MLEP/index.html

**Operating systems:** Linux/Mac/Windows

**Programming language:** R and C

**Other requirements:** R version≥2.14

**License:** GPL≥2

**Any restrictions to use by non-academics:** None

## Availability of supporting data

The data set supporting the results of this article is available at http://www.stat.math.keio.ac.jp/∼sugaya/PIA/MLEP/index.html.

## Competing interests

The author declares no competing interests.

## Author’s contributions

YS implemented the software and prepared the manuscript.

## Supplementary Material

Additional file 1 Algorithm for likelihood polynomial of penetrance parameters. Click here for file

Additional file 2 **Figure S1.** Perspective plots of log likelihood surface for the simulation study of the penetrance model, 0.950, 0.700, and 0.000. The log likelihood surfaces are plotted for 30 cases of disease allele frequencies of the penetrance model, 0.950, 0.700, and 0.000. Fixing *γ* at its estimate evaluated under the constraint 0 ≤* γ *≤ 0.01, each log likelihood surface is drawn on a limited region *α *≥* β*. Click here for file

Additional file 3 **Table S1.** Summary of maximum likelihood estimates of penetrance parameters for the simulation study of the penetrance model, 0.990, 0.900, and 0.000. The pedigree structure, number of marker alleles, marker allele frequencies, and recombination fractions are identical to those used in the simulation for the penetrance model, 0.950, 0.700, and 0.000, while penetrance parameters are assumed to be 0.990, 0.900, and 0.000. Six pedigree datasets with 50 pedigrees are generated, assuming disease allele frequencies to be 0.0001, 0.001, 0.01, 0.1, and 0.2. For each datasets, the likelihood polynomial is evaluated, assuming the frequency to be 0.0001, 0.001, 0.01, 0.1, 0.25, and 0.5, and the maximum likelihood estimates are evaluated under the constraint 0 ≤* γ *≤ 0.01. Click here for file

Additional file 4 **Table S2.** Summary of LOD scores for the simulation study of the penetrance model, 0.990, 0.900, and 0.000. LOD scores are evaluated for 30 cases of disease allele frequencies for three penetrance models; true penetrance model (0.990, 0.900, and 0.000), dominant model (0.999, 0.999, and 0.000),and MLE model (the estimates are listed in Supplementary Table S1). Click here for file

Additional file 5 **Figure S2.** Perspective plots of log likelihood surface for the simulation study of the penetrance model, 0.990, 0.900, and 0.000. The log likelihood surfaces are plotted for 30 cases of disease allele frequencies of the penetrance model, 0.990, 0.900, and 0.000. Fixing *γ* at its constrained estimate under the 0 ≤* γ *≤ 0.01, each log likelihood surface is drawn on a limited region *α *≥* β*. Click here for file
