# Supplementary material for: MLEP: an R package for exploring the maximum likelihood estimates of penetrance parameters
Source: BMC Res Notes. 2012 Aug 28;5:465. doi: 10.1186/1756-0500-5-465 (PMC3537736; doi:10.1186/1756-0500-5-465)
Supplement: Additional file 1 — Algorithm for likelihood polynomial of penetrance parameters. [file 1756-0500-5-465-S1.pdf]

Supplementary Methods

MLEP: an R package for exploring the maximum likelihood estimates of penetrance parameters

Yuki Sugaya*[1]

[1]School of Fundamental Science and Technology, Keio University, Yokohama, Japan

Email: Yuki Sugaya*- sugaya@stat.math.keio.ac.jp;

*Corresponding author

Notation

Let V be a set of individuals in a pedigree. For various sets within a pedigree, we hereafter use the following notations. A set of founders is denoted by $\{v;\ v \in V,\ \pi(v) = \emptyset\}$, where $\pi(v)$ is the set of the parents of individual v. The notation $\Pi(v)$ is used for the set of ancestors of individual v. The set of siblings of $v \in V$ is $\sigma(v) = \{u;\ u \in V,\ u \neq v,\ \pi(v) \cap \pi(u) \neq \emptyset\}$. Any individuals in $\sigma(v) \cup \{v\}$ without a descendant in V are referred to as terminal sibling sets.

Likelihood

The likelihood is the probability $p_{\tilde{\boldsymbol{\theta}}}(a_V) = P_{\tilde{\boldsymbol{\theta}}}(\{A_V = a_V\})$ that random variables for affected status A_V assume the values a_V, where $A_V = (A_v)_{v \in V}$ and $a_V = (a_v)_{v \in V}$. The notation defined for an individual is also extensively used for a set of individuals throughout this paper. Here, a_v is assigned the value $affected$ if it is affected; otherwise, it is designated $unaffected$. If the affected status is unknown, a_v is designated NA (not available). The parameter vector $\tilde{\boldsymbol{\theta}}$ comprises the penetrance parameters as conditional probabilities given the disease locus genotype, G_v, such that $\alpha = P(A_v = affected|G_v = A/A)$, $\beta = P(A_v = affected|G_v = A/a)$, and $\gamma = P(A_v = affected|G_v = a/a)$, where A and a are the disease and normal alleles, respectively and A/A, A/a, and a/a represent the possible genotypes of the disease locus.

Algorithm

For an individual v in a terminal sibling set, the likelihood can be expressed (using v as a pivot variable) as

$$\begin{aligned} L(\tilde{\boldsymbol{\theta}}) &= \sum_{g_v} p_{\tilde{\boldsymbol{\theta}}}(a_v|g_v, a_{V\backslash\{v\}})\ p_{\tilde{\boldsymbol{\theta}}}(g_v, a_{V\backslash\{v\}}) \\ &= \sum_{g_v} p_{\tilde{\boldsymbol{\theta}}}(a_v|g_v)\ p_{\tilde{\boldsymbol{\theta}}}(g_v, a_{\Pi(v)}), \end{aligned}$$

where $V\backslash\{v\} = \Pi(v)$ and g_v represents a disease locus genotype of individual v . The likelihood is described by the following recursive formula: The probability $p_{\tilde{\boldsymbol{\theta}}}(g_v, a_{\Pi(v)})$ appearing in the right-hand side of the above formula is written in terms of the probabilities $p_{\tilde{\boldsymbol{\theta}}}(d_{p^0}, a_{\Pi(p^0)})$ and $p_{\tilde{\boldsymbol{\theta}}}(d_{p^1}, a_{\Pi(p^1)})$ of the parents $p = \pi(v) = (p^0, p^1)$ as

$$\begin{aligned} p_{\tilde{\boldsymbol{\theta}}}(g_v, a_{\Pi(v)}) = \sum_{g_p} \Big\{ & p_{\tilde{\boldsymbol{\theta}}}(a_{p^0}|g_{p^0})\ p_{\tilde{\boldsymbol{\theta}}}(a_{p^1}|g_{p^1})\ p(g_v|g_p)\ s(g_p) \\ & \times p_{\tilde{\boldsymbol{\theta}}}(g_{p^0}, a_{\Pi(p^0)})\ p_{\tilde{\boldsymbol{\theta}}}(g_{p^1}, a_{\Pi(p^1)}) \Big\}, \end{aligned} \tag{1}$$

where

$$s(g_p) = \prod_{u \in \sigma(v)} \Big\{ \sum_{g_u} p_{\tilde{\boldsymbol{\theta}}}(a_u|g_u)\ p(g_u|g_p) \Big\}. \tag{2}$$

After repeatedly applying formula (1) with p^0 or p^1 as the pivot variable, the status of $\Pi(v)$ becomes empty, implying that v is a founder. We then set $p(g_v, a_\emptyset) = p(g_v) = p(g_v^0)p(g_v^1)$, where the probabilities $p(g_v^0)$ and $p(g_v^1)$ take value f_A or $1 - f_A$. Note that the paternal allele and maternal allele are denoted by g_v^0 and g_v^1, and the disease allele frequency by f_A. The likelihood is therefore evaluated by backward tracing to the terminal sibling. The probability $\sum_{g_{v_0}} p_{\tilde{\boldsymbol{\theta}}}(a_{v_0}|g_{v_0})\ p(g_{v_0}|g_p)$ in the right-hand side of formula (2) can be replaced with

$$\sum_{g_{v_0}} p_{\tilde{\boldsymbol{\theta}}}(a_{v_0}|g_{v_0})\ p(g_{v_0}|g_p)\ p_{\tilde{\boldsymbol{\theta}}}(a_{V_1}|g_{v_0})$$

if the individual v_0 is connected to a set of ancestors V_1 of the other terminal sibling set. The conditional likelihood $p_{\tilde{\boldsymbol{\theta}}}(a_{V_1}|d_{v_0})$ can also be computed from the same algorithm by assuming that v_0 is a founder of V_1 with the genotype g_{v_0}. The above procedure can also be applied when multiple terminal sibling sets exist in the given pedigree.

The probability $p(g_v|g_p)$, expressed as a product of the allele inheritance probabilities, is $p(g_v^0|g_{p^0})p(g_v^1|g_{p^1})$, where each probability is one of 1, 0.5, or 0. Since the founder likelihood also assumes a scalar value, f_A or $1-f_A$, the likelihood $L(\tilde{\boldsymbol{\theta}})$ is clearly a polynomial of the penetrance parameters. Since the parameters are produced by $p_{\tilde{\boldsymbol{\theta}}}(a_{p^0}|g_{p^0})$ and $p_{\tilde{\boldsymbol{\theta}}}(a_{p^1}|g_{p^1})$, the maximum summation of the powers i, j, and k

equals the number of individuals whose disease status (affected or unaffected) is known. In the algorithm, only the coefficients c_{ijk} of the polynomial

$$p_{\tilde{\boldsymbol{\theta}}}(d_v, a_{\Pi(v)}) = \sum_{i,j,k} c_{ijk} \alpha^i \beta^j \gamma^k,$$

are inherited, where the indexes i, j, and k run over from 0 to N subject to the constraint $max(i+j+k) = N$. Here, N is the number of individuals whose disease status is known.
