# Supplementary material for: MLEP: an R package for exploring the maximum likelihood estimates of penetrance parameters
Source: BMC Res Notes. 2012 Aug 28;5:465. doi: 10.1186/1756-0500-5-465 (PMC3537736; doi:10.1186/1756-0500-5-465)

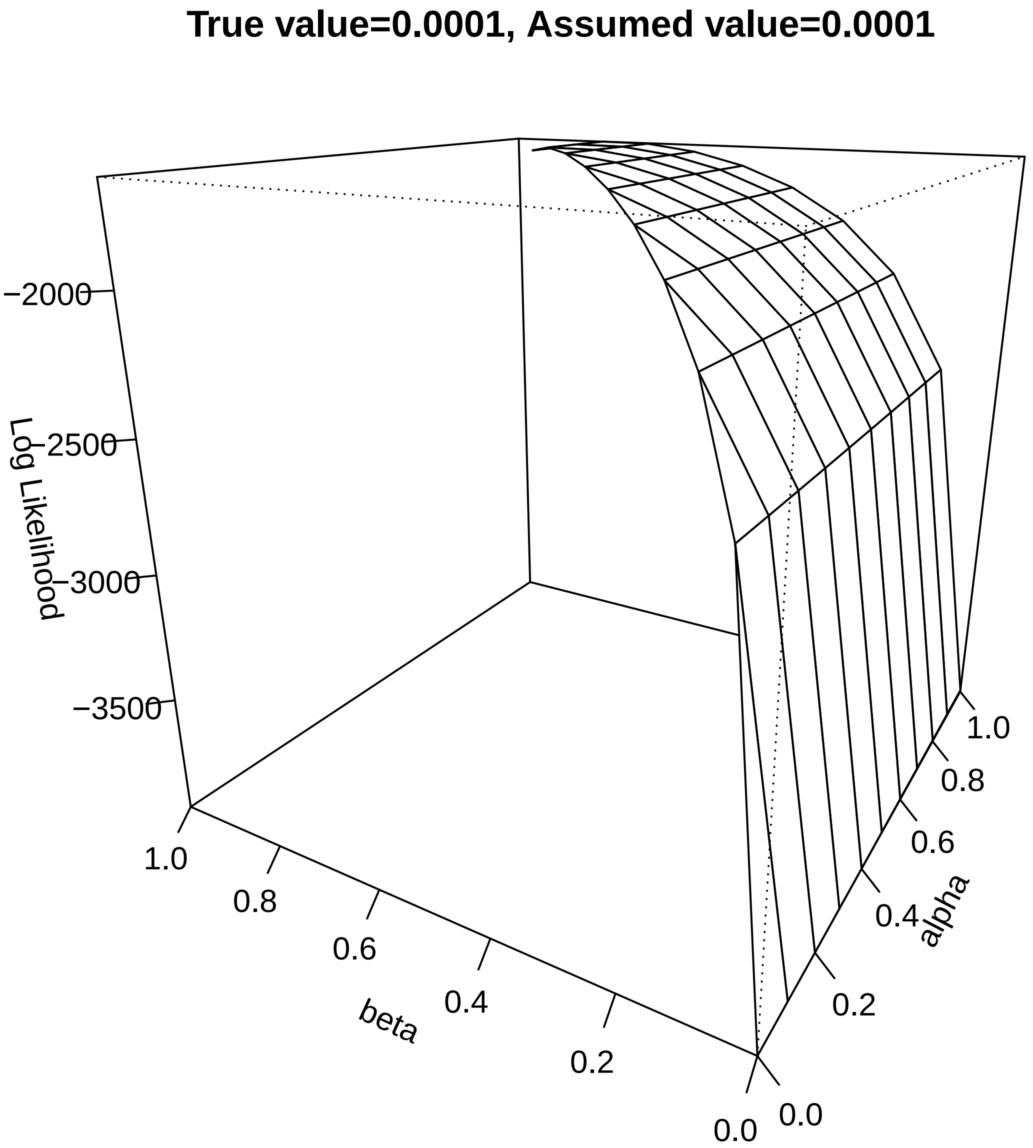
True value=0.0001, Assumed value=0.0001
Log Likelihood
−2000
−2500
−3000
−3500
beta
1.0
0.8
0.6
0.4
0.2
0.0
alpha
0.0
0.2
0.4
0.6
0.8
1.0

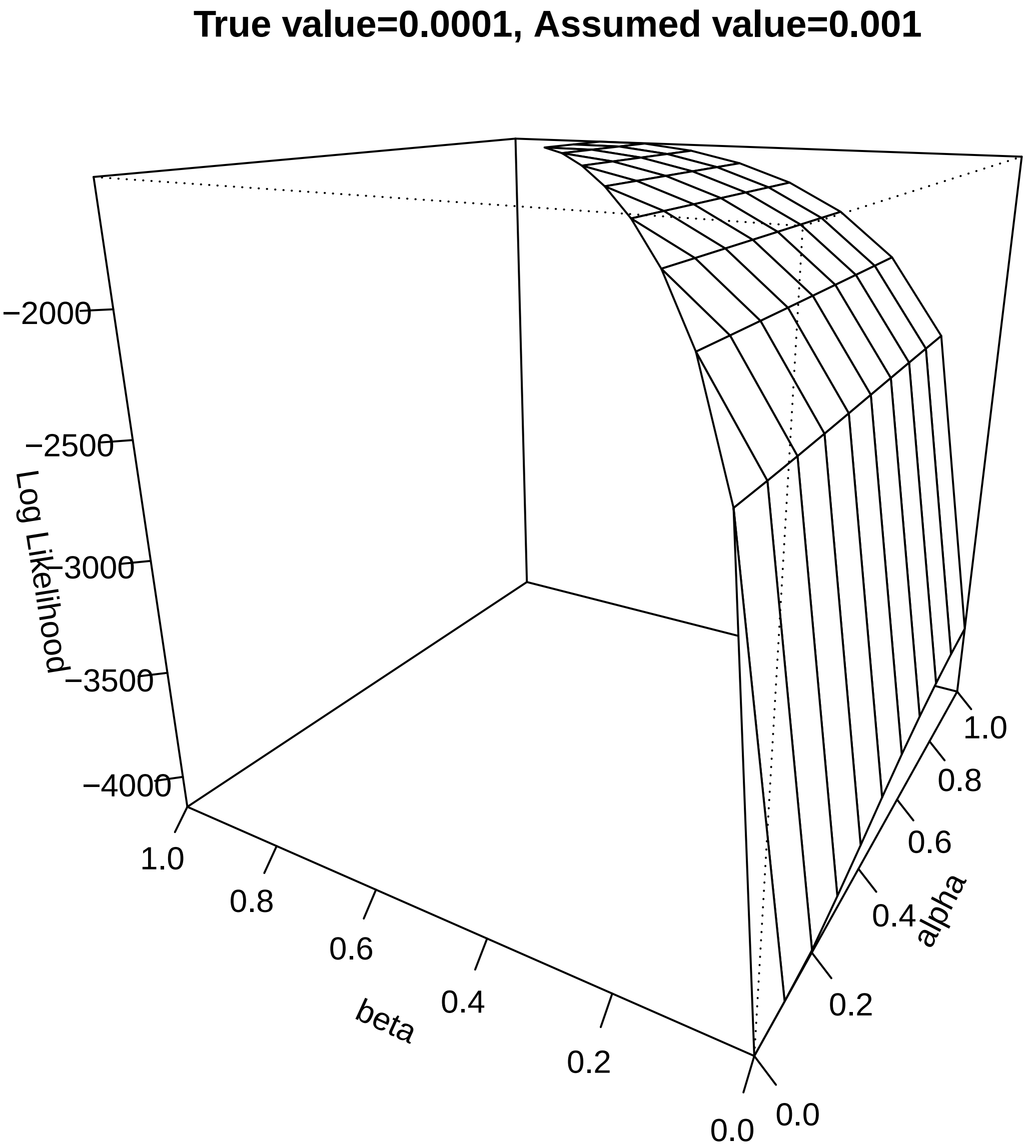

True value=0.0001, Assumed value=0.001
Log Likelihood
−2000
−2500
−3000
−3500
−4000
beta
1.0
0.8
0.6
0.4
0.2
0.0
alpha
0.0
0.2
0.4
0.6
0.8
1.0

True value=0.0001, Assumed value=0.01

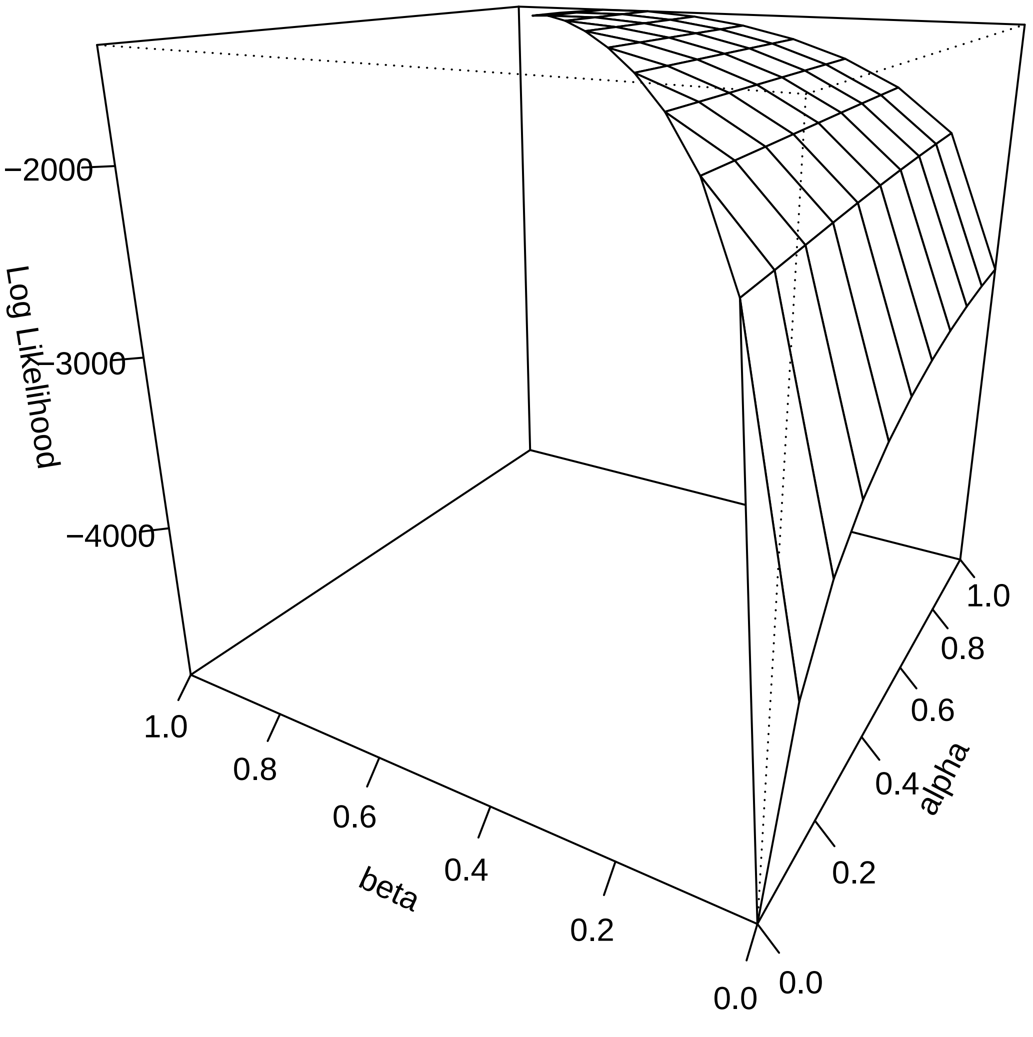

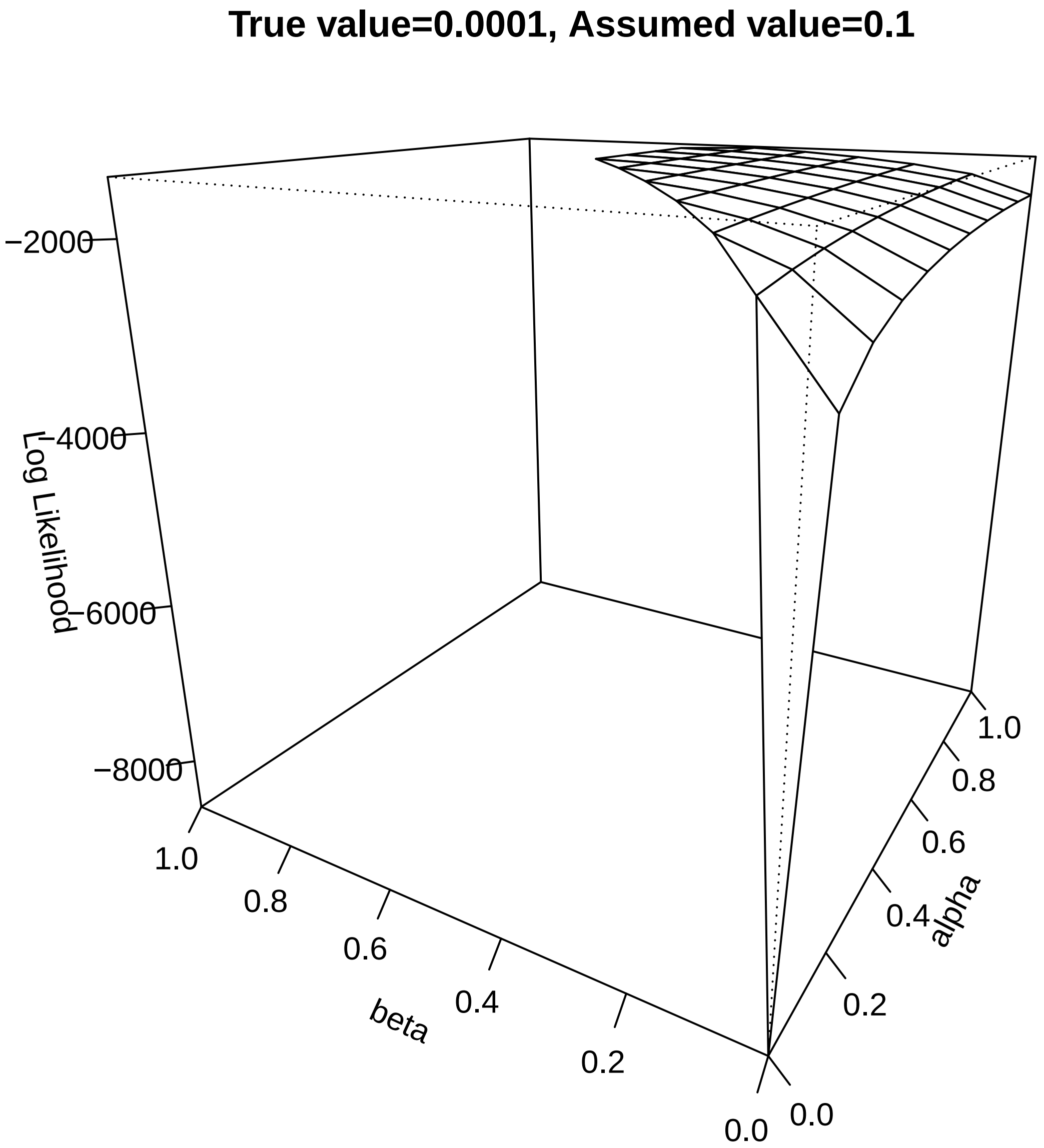
True value=0.0001, Assumed value=0.1
Log Likelihood
−2000
−4000
−6000
−8000
beta
1.0
0.8
0.6
0.4
0.2
0.0
alpha
0.0
0.2
0.4
0.6
0.8
1.0

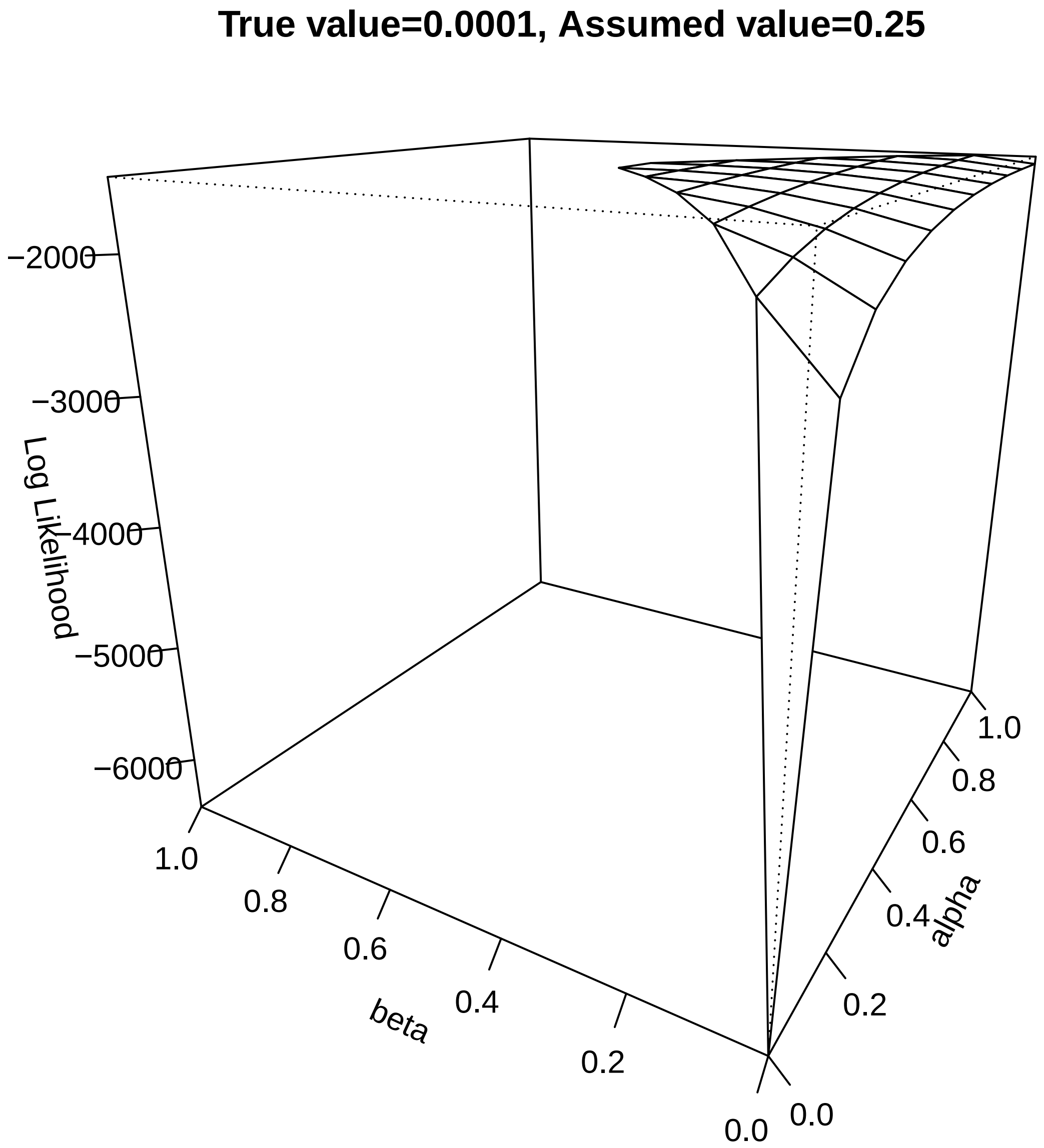
True value=0.0001, Assumed value=0.25
Log Likelihood
−2000
−3000
−4000
−5000
−6000
beta
1.0
0.8
0.6
0.4
0.2
0.0
alpha
0.0
0.2
0.4
0.6
0.8
1.0

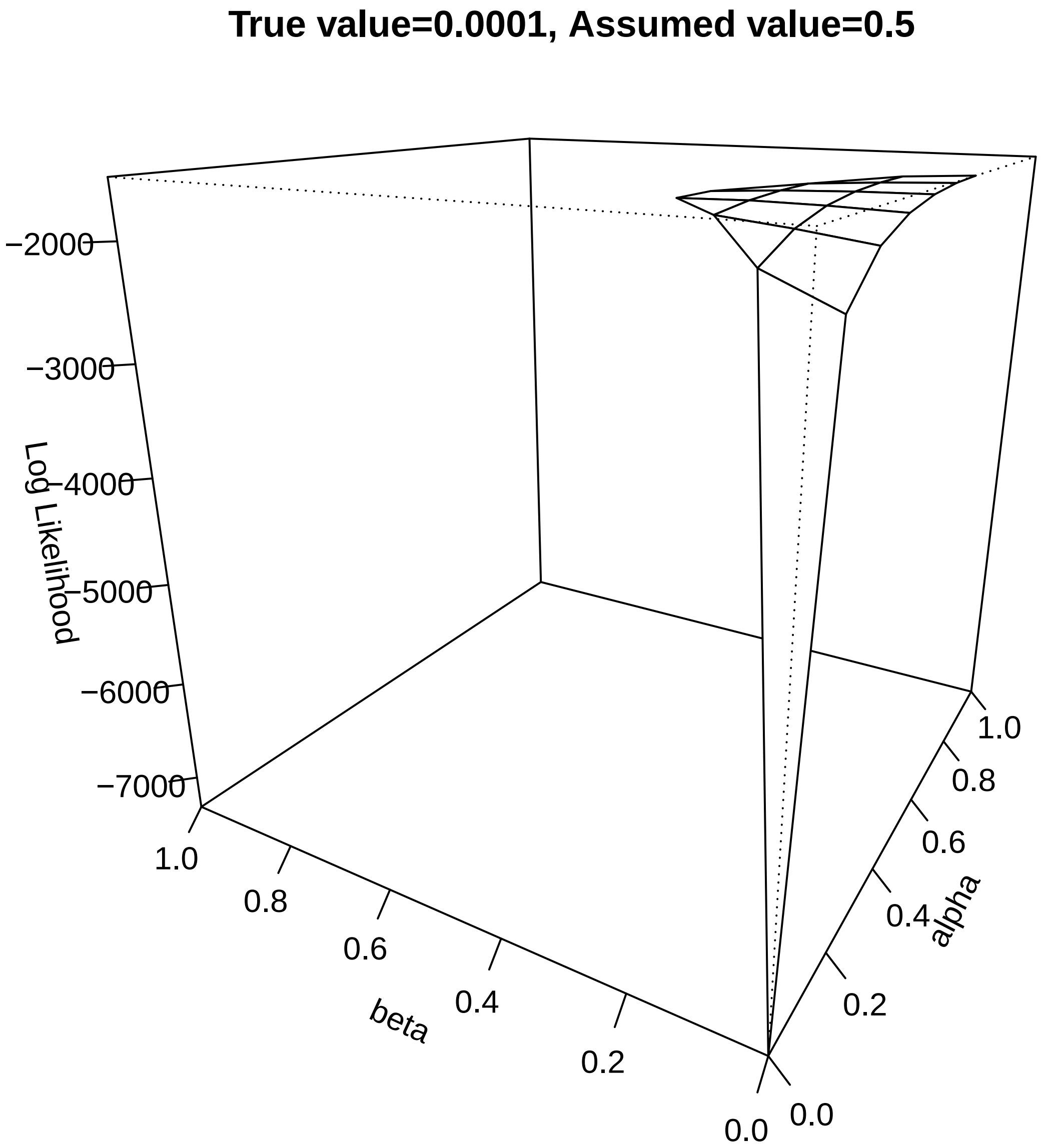
True value=0.0001, Assumed value=0.5
−2000
−3000
−4000
−5000
−6000
−7000
Log Likelihood
1.0
0.8
0.6
0.4
0.2
0.0
beta
0.0
0.2
0.4
0.6
0.8
1.0
alpha

True value=0.001, Assumed value=0.0001

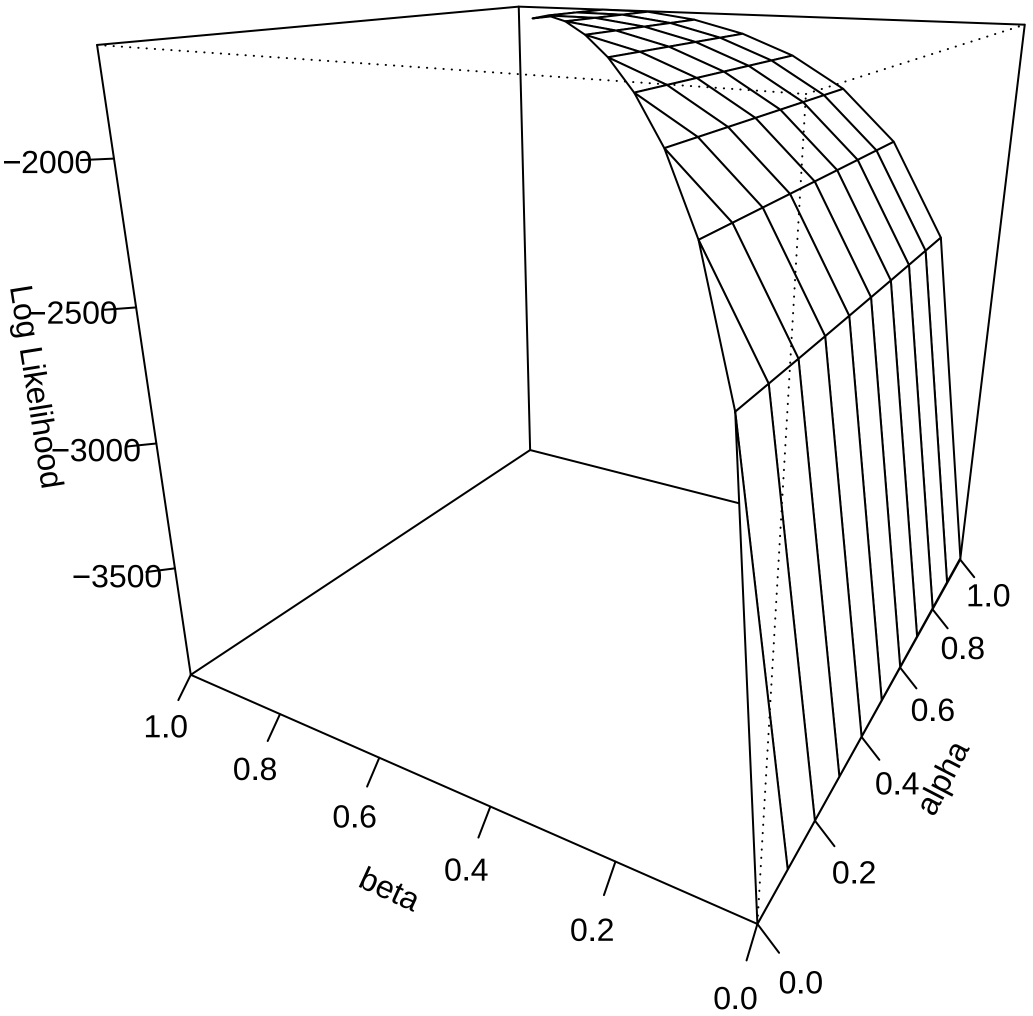

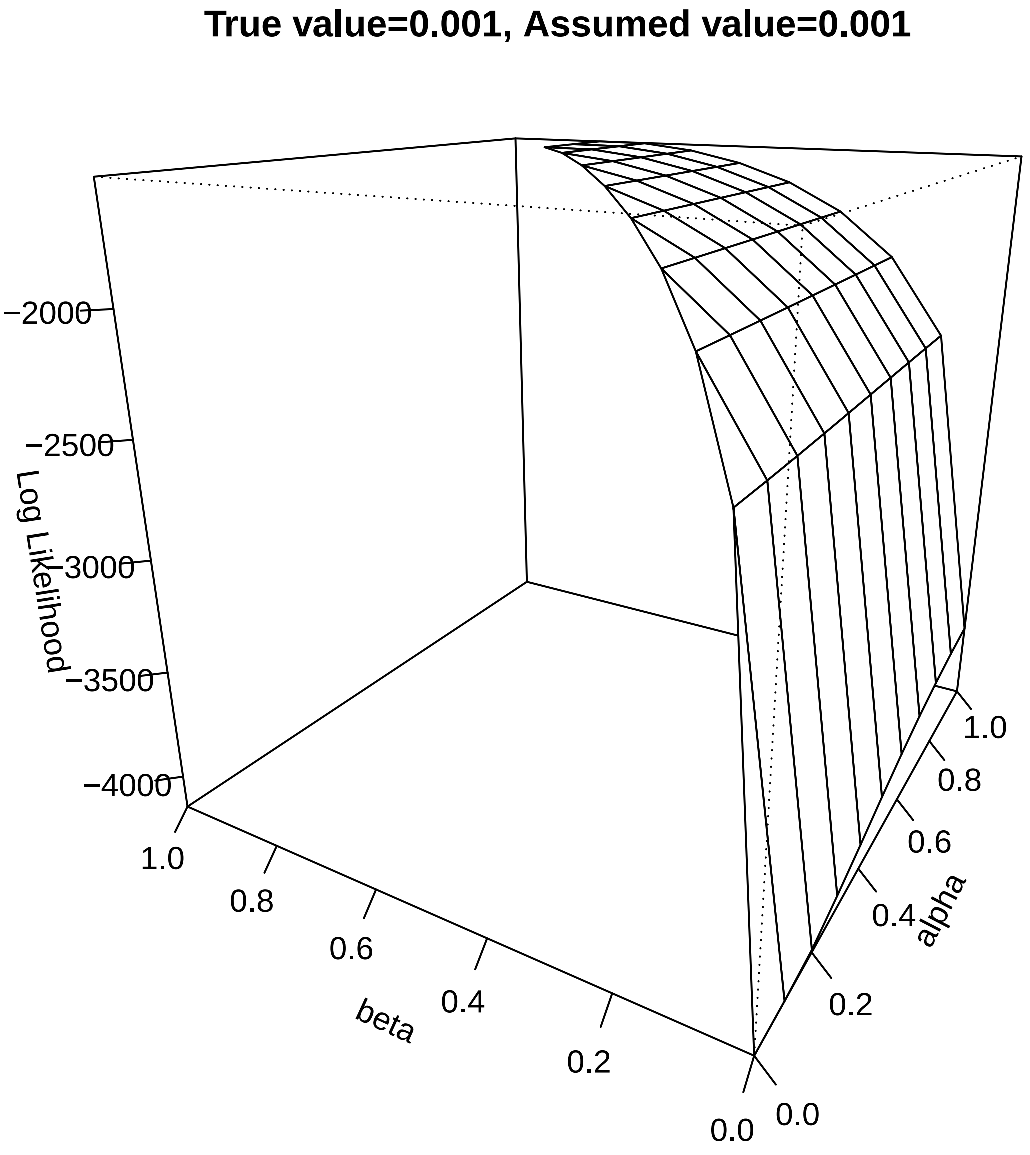
True value=0.001, Assumed value=0.001
−2000
−2500
−3000
−3500
−4000
Log Likelihood
1.0
0.8
0.6
0.4
0.2
0.0
beta
0.0
0.2
0.4
0.6
0.8
1.0
alpha

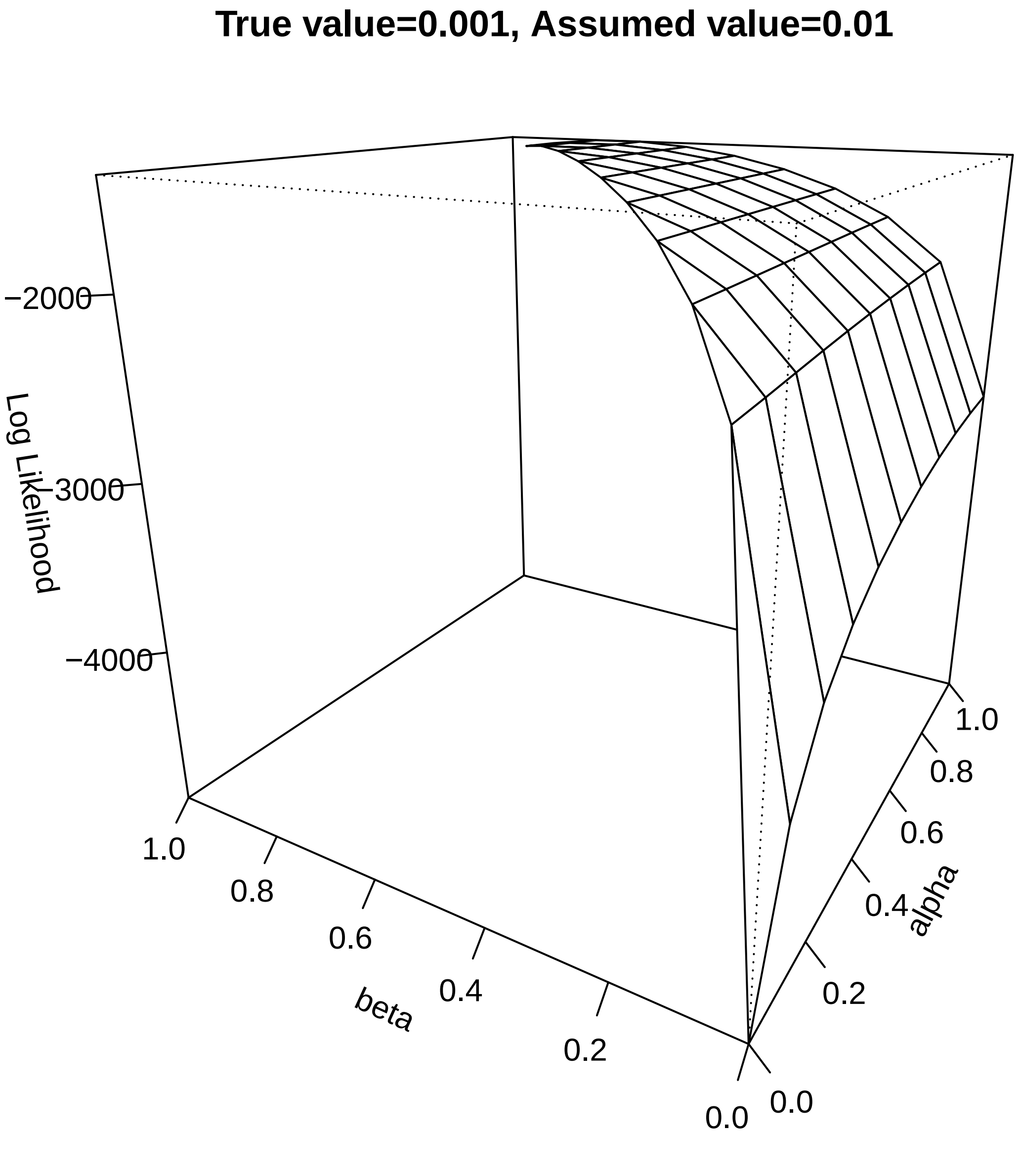
True value=0.001, Assumed value=0.01
Log Likelihood
−2000
−3000
−4000
beta
1.0
0.8
0.6
0.4
0.2
0.0
alpha
0.0
0.2
0.4
0.6
0.8
1.0

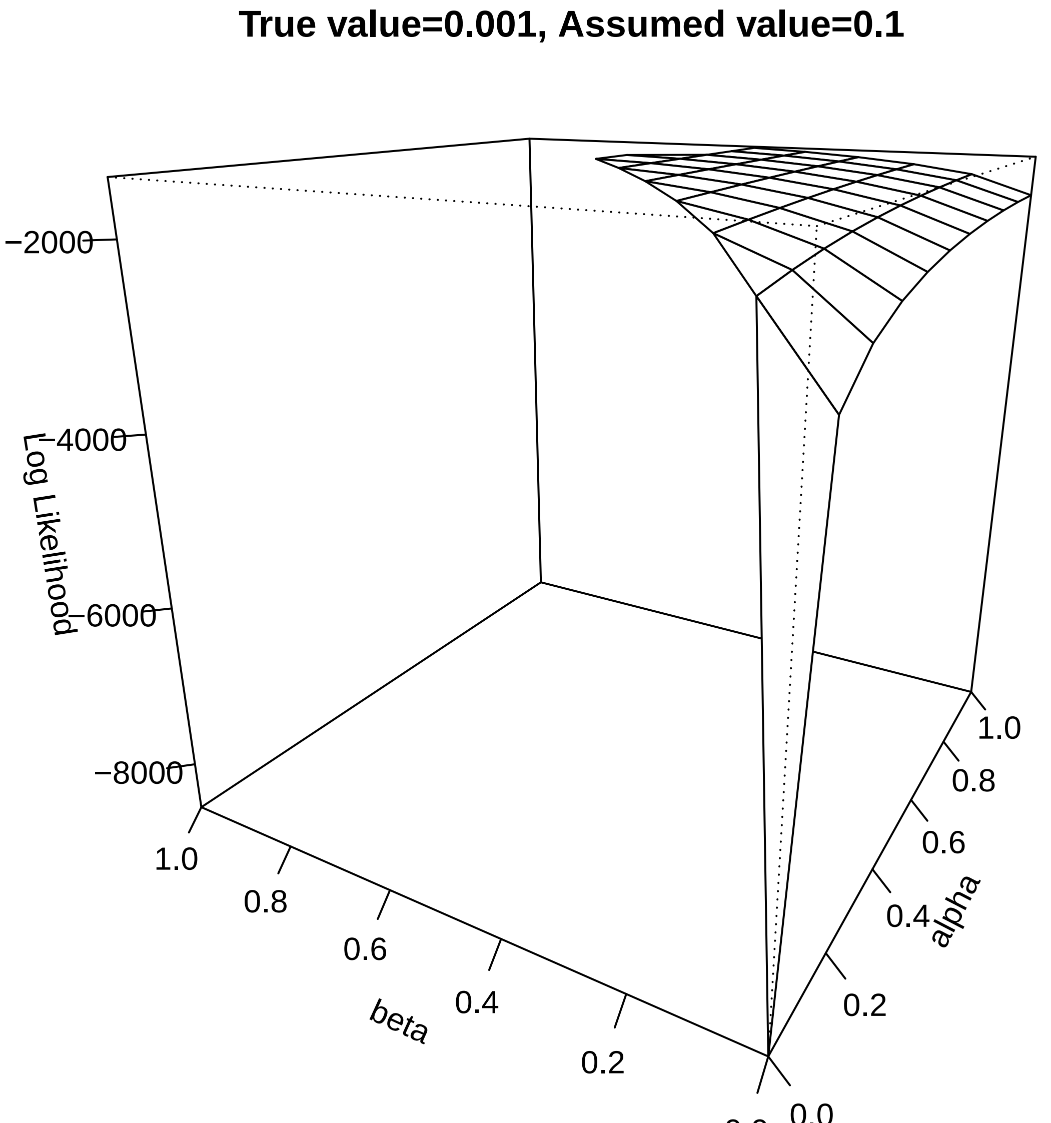
True value=0.001, Assumed value=0.1
Log Likelihood
−2000
−4000
−6000
−8000
beta
1.0
0.8
0.6
0.4
0.2
0.0
alpha
0.0
0.2
0.4
0.6
0.8
1.0

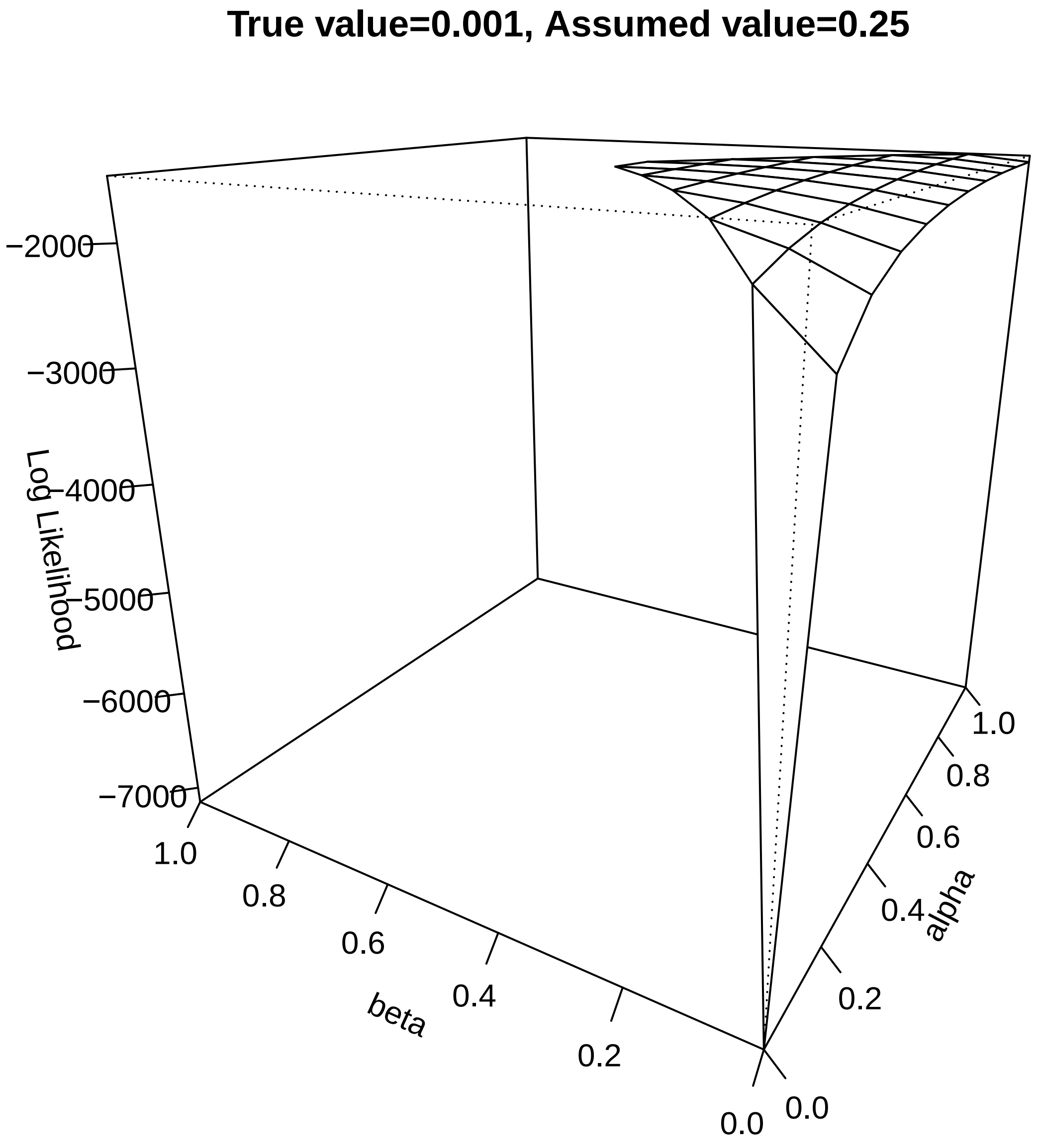
True value=0.001, Assumed value=0.25
−2000
−3000
−4000
−5000
−6000
−7000
Log Likelihood
1.0
0.8
0.6
0.4
0.2
0.0
beta
0.0
0.2
0.4
0.6
0.8
1.0
alpha

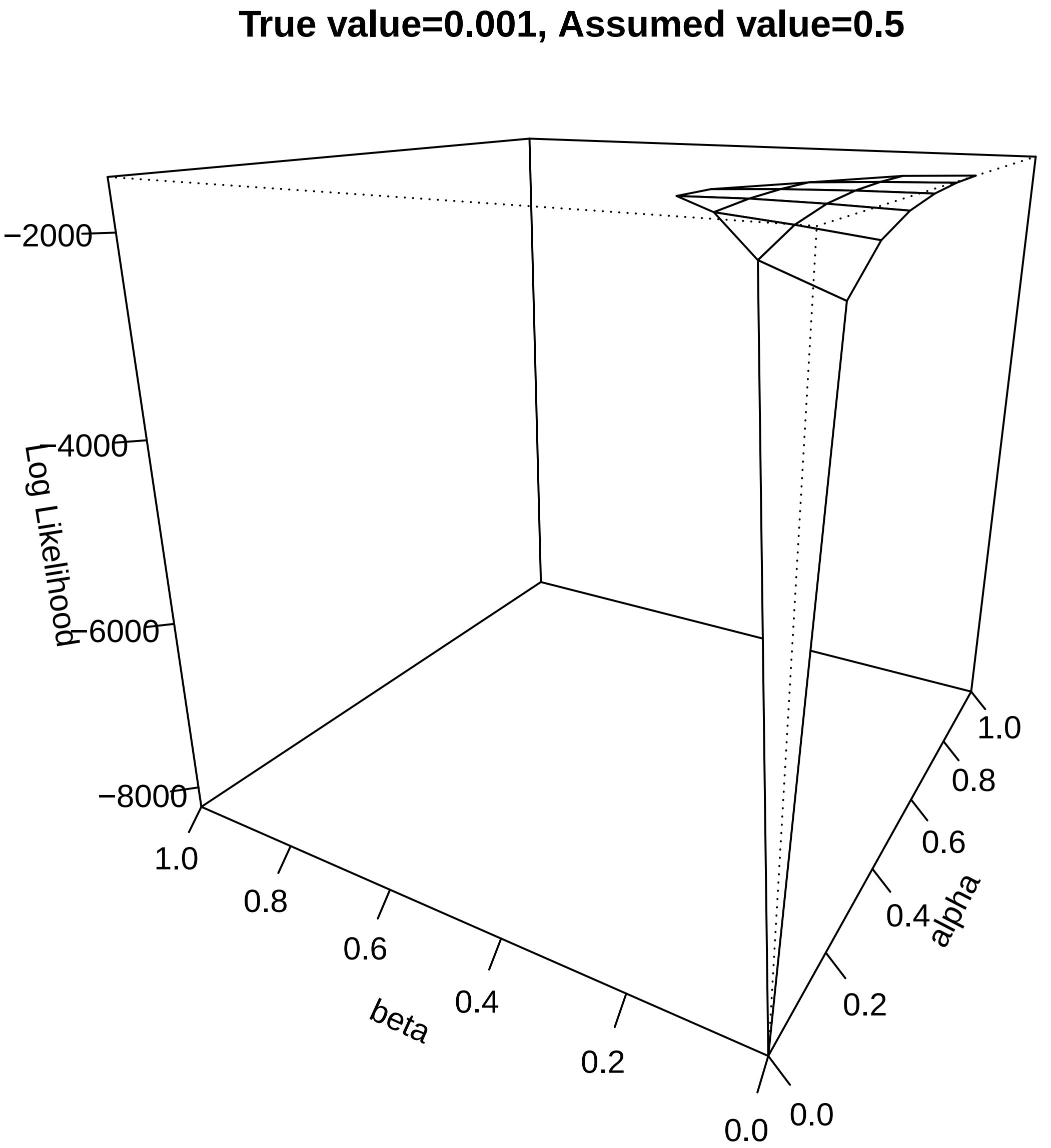
True value=0.001, Assumed value=0.5
Log Likelihood
−2000
−4000
−6000
−8000
beta
1.0
0.8
0.6
0.4
0.2
0.0
alpha
0.0
0.2
0.4
0.6
0.8
1.0

True value=0.01, Assumed value=0.0001

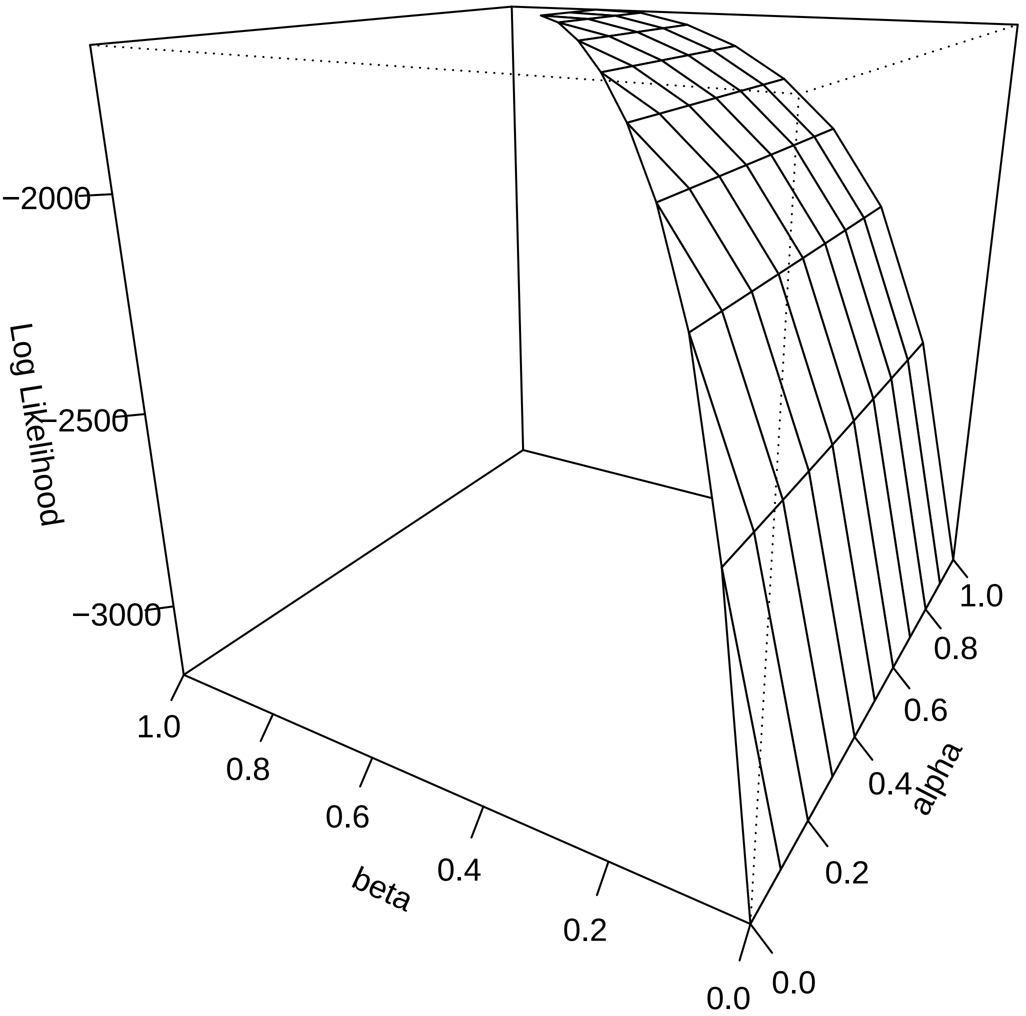

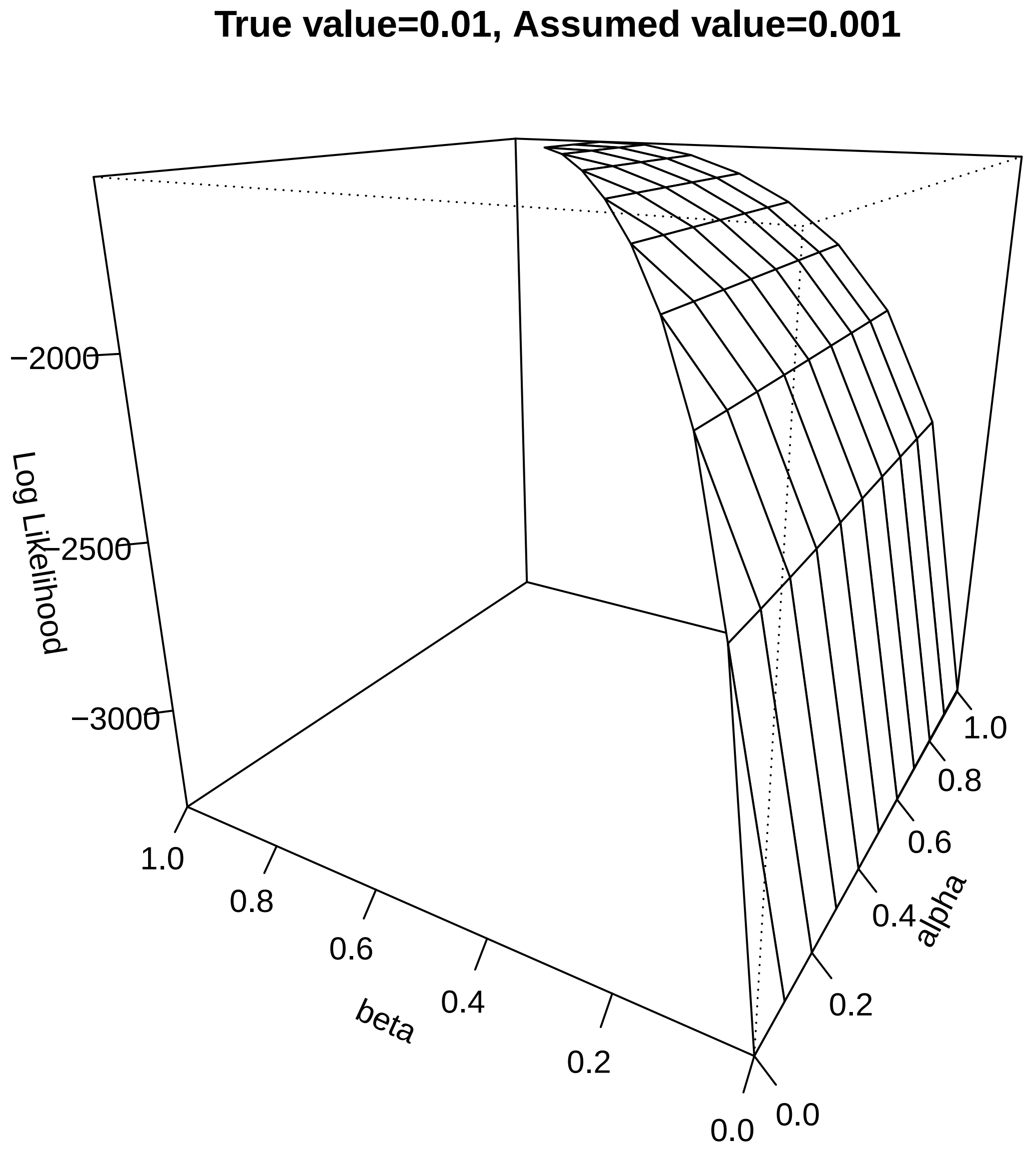
True value=0.01, Assumed value=0.001
Log Likelihood
−2000
−2500
−3000
beta
1.0
0.8
0.6
0.4
0.2
0.0
alpha
0.0
0.2
0.4
0.6
0.8
1.0

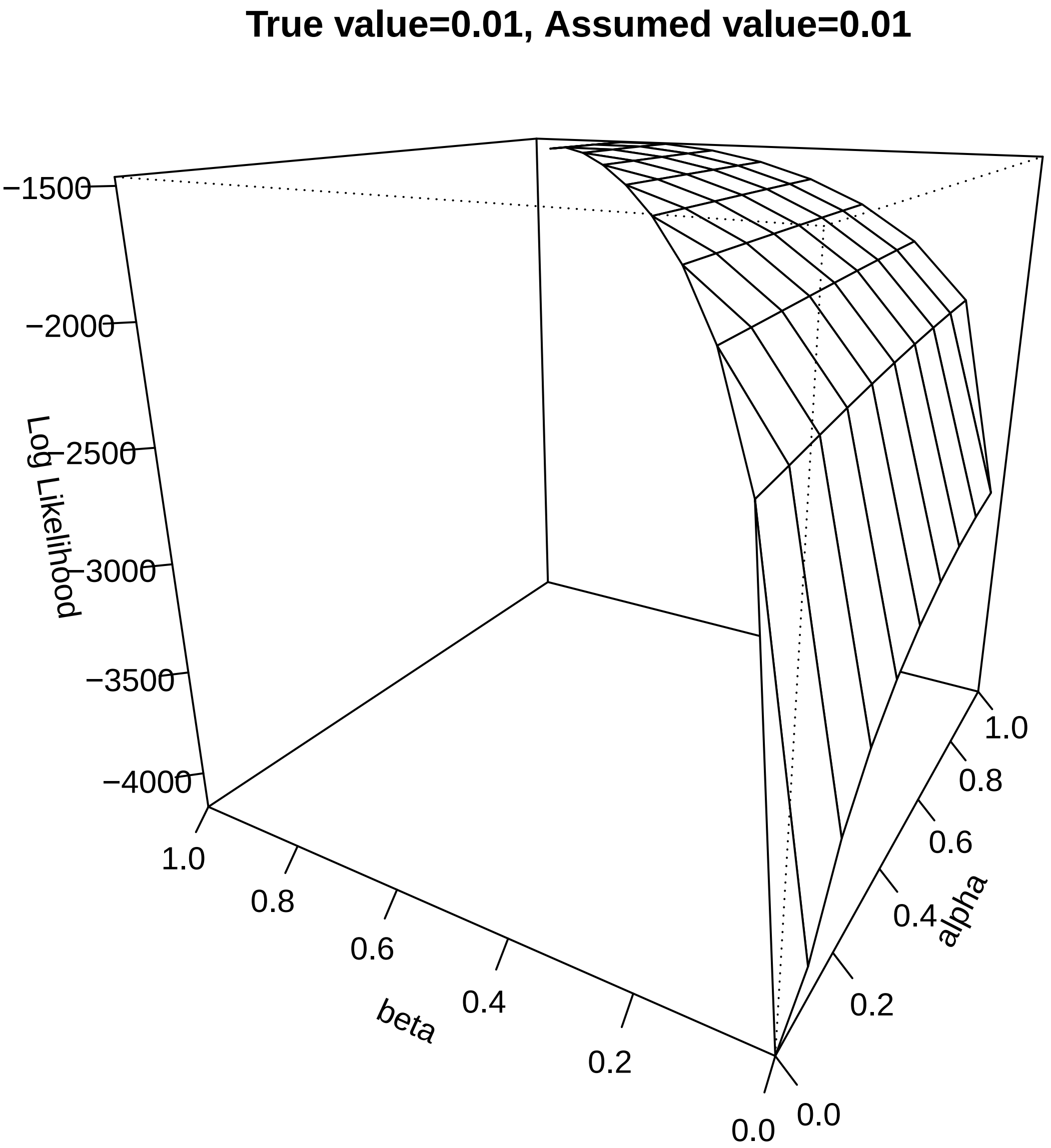
True value=0.01, Assumed value=0.01
Log Likelihood
−1500
−2000
−2500
−3000
−3500
−4000
beta
1.0
0.8
0.6
0.4
0.2
0.0
alpha
0.0
0.2
0.4
0.6
0.8
1.0

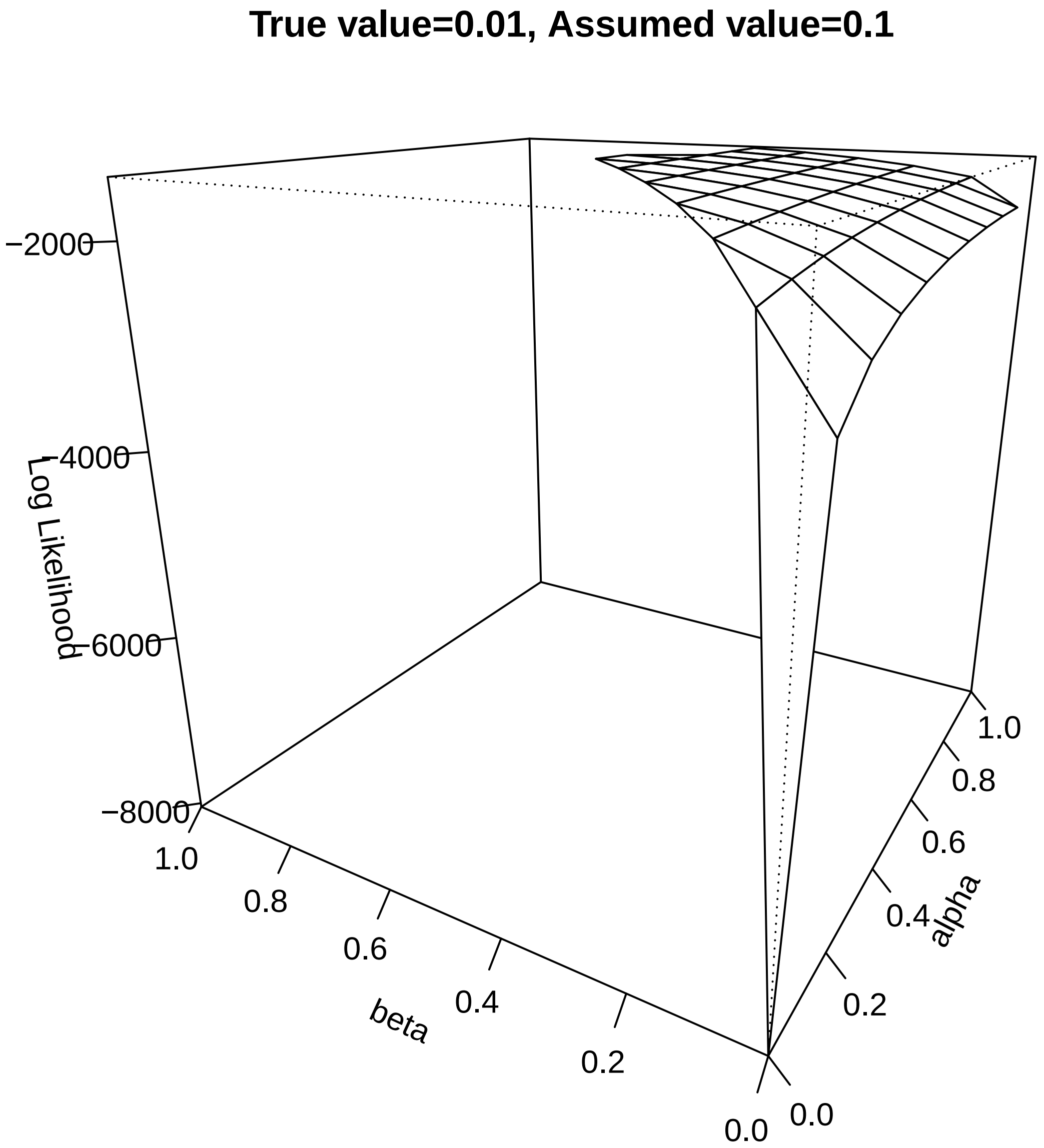

True value=0.01, Assumed value=0.1
−2000
−4000
−6000
−8000
Log Likelihood
1.0
0.8
0.6
0.4
0.2
0.0
beta
0.0
0.2
0.4
0.6
0.8
1.0
alpha

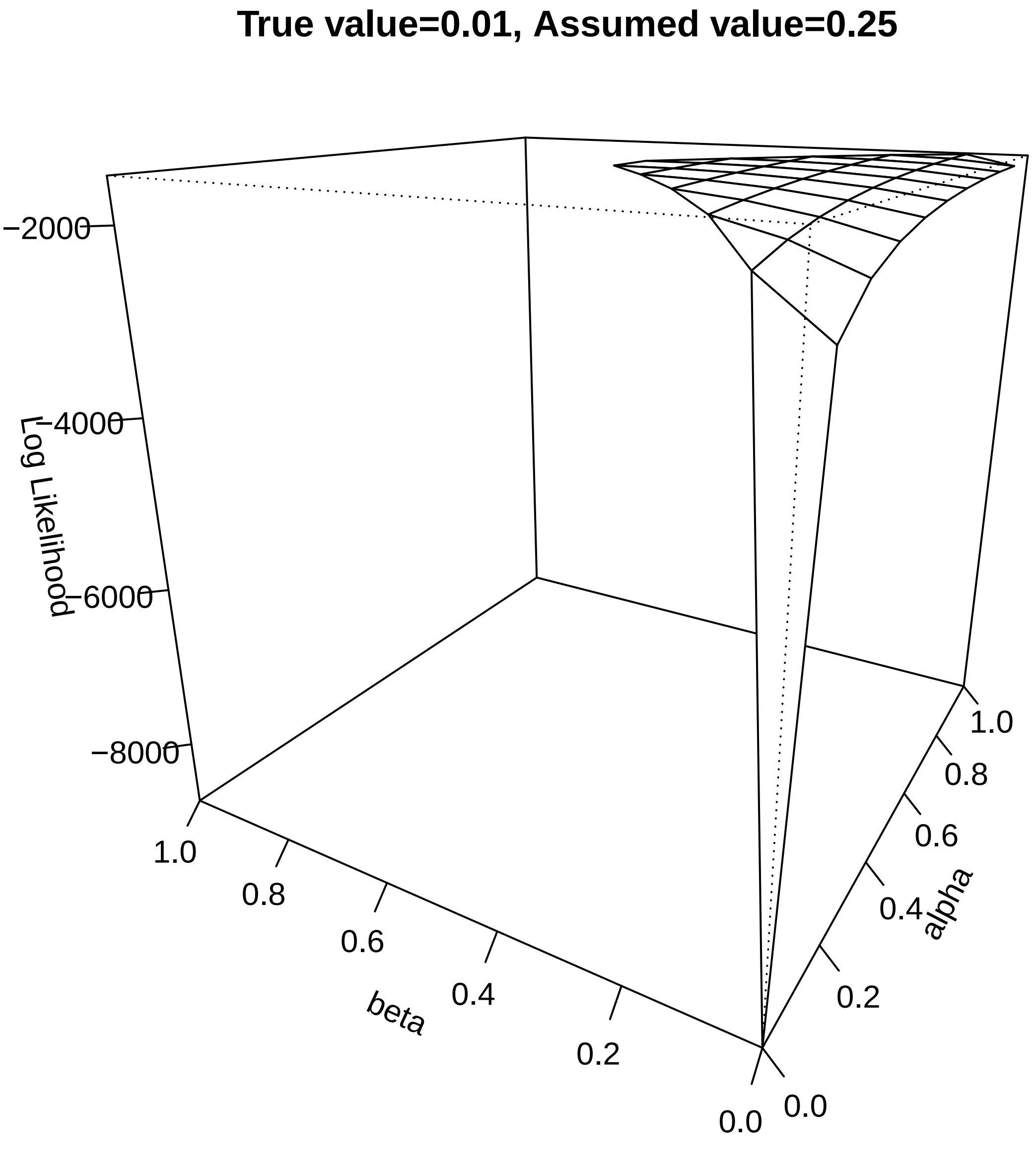

True value=0.01, Assumed value=0.25
Log Likelihood
−2000
−4000
−6000
−8000
beta
1.0
0.8
0.6
0.4
0.2
0.0
alpha
0.0
0.2
0.4
0.6
0.8
1.0

True value=0.01, Assumed value=0.5

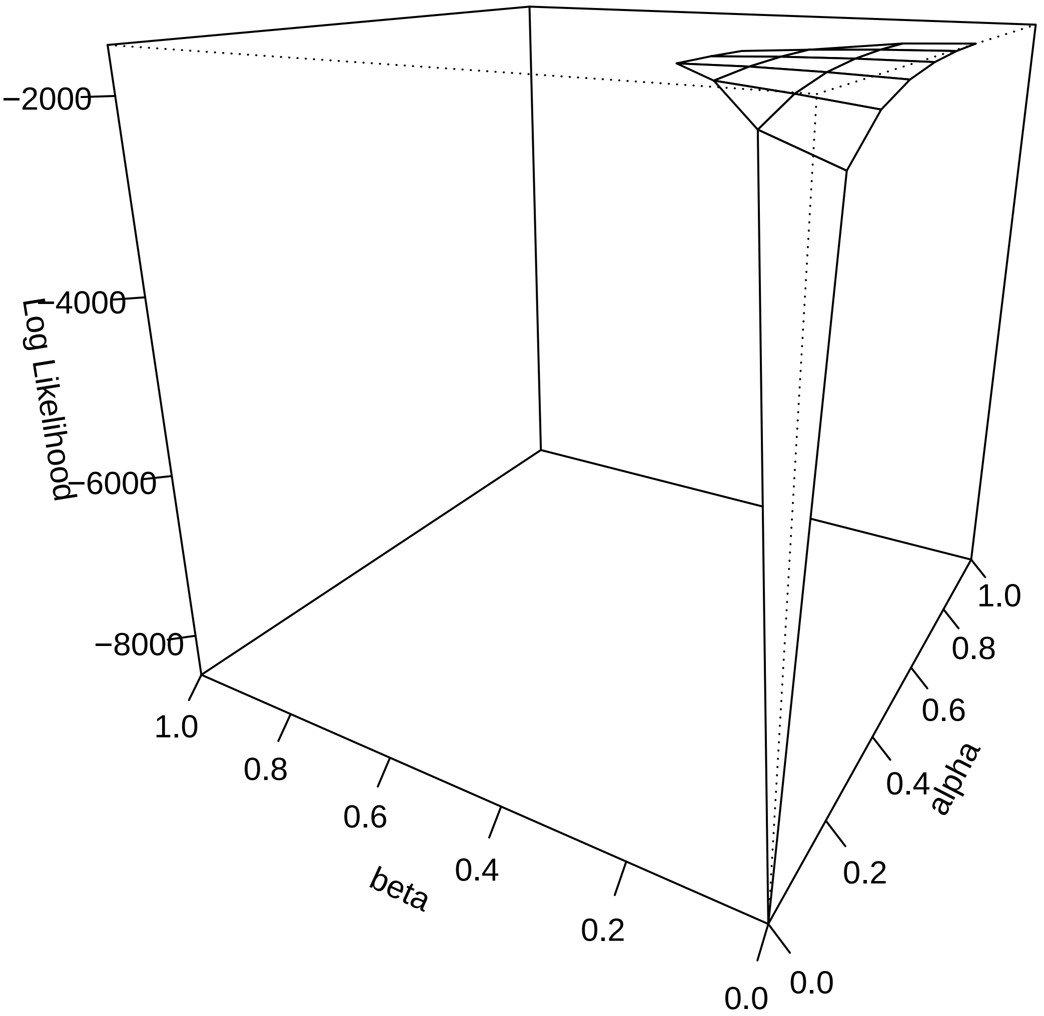

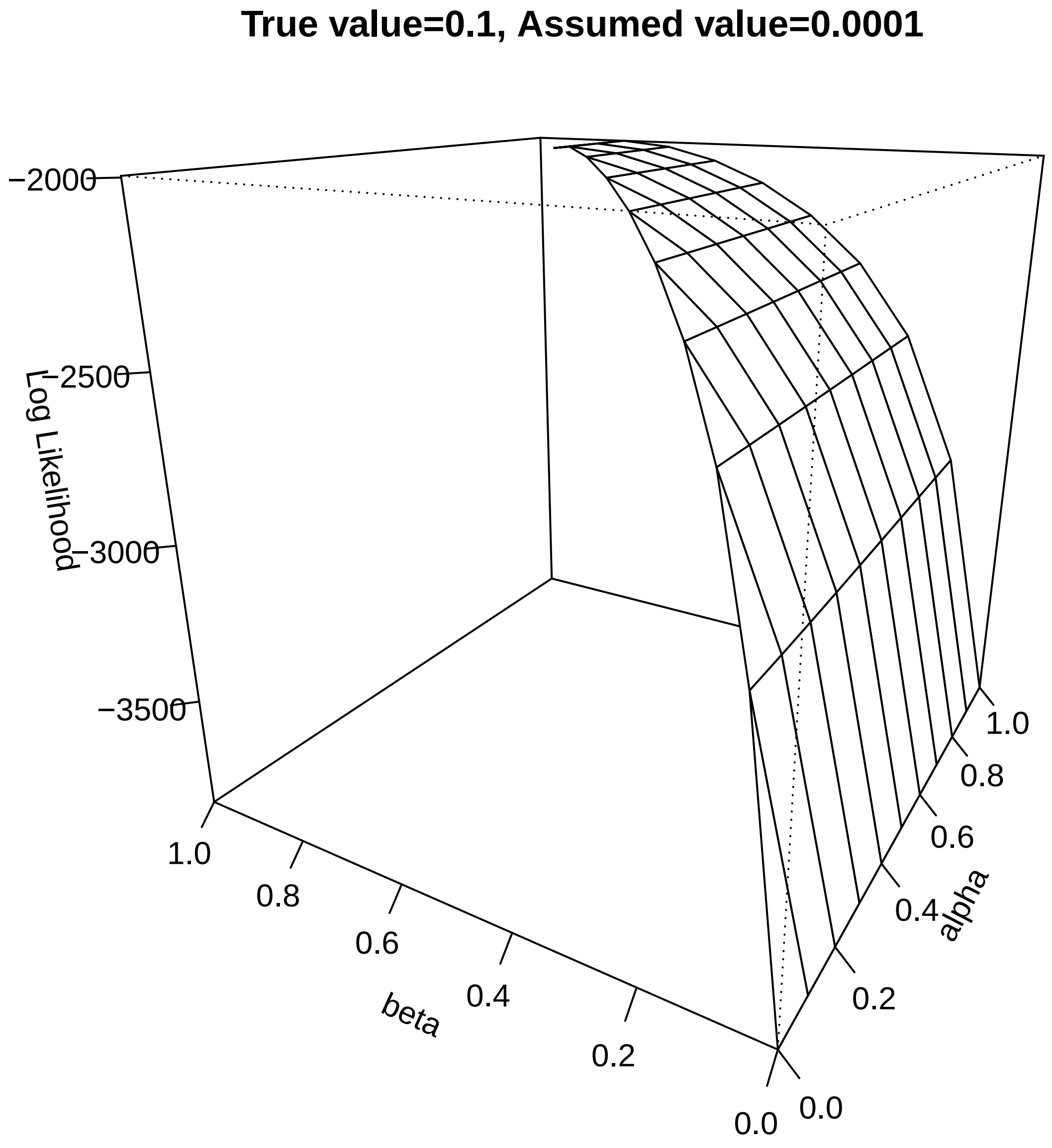

True value=0.1, Assumed value=0.0001
Log Likelihood
−2000
−2500
−3000
−3500
beta
1.0
0.8
0.6
0.4
0.2
0.0
alpha
0.0
0.2
0.4
0.6
0.8
1.0

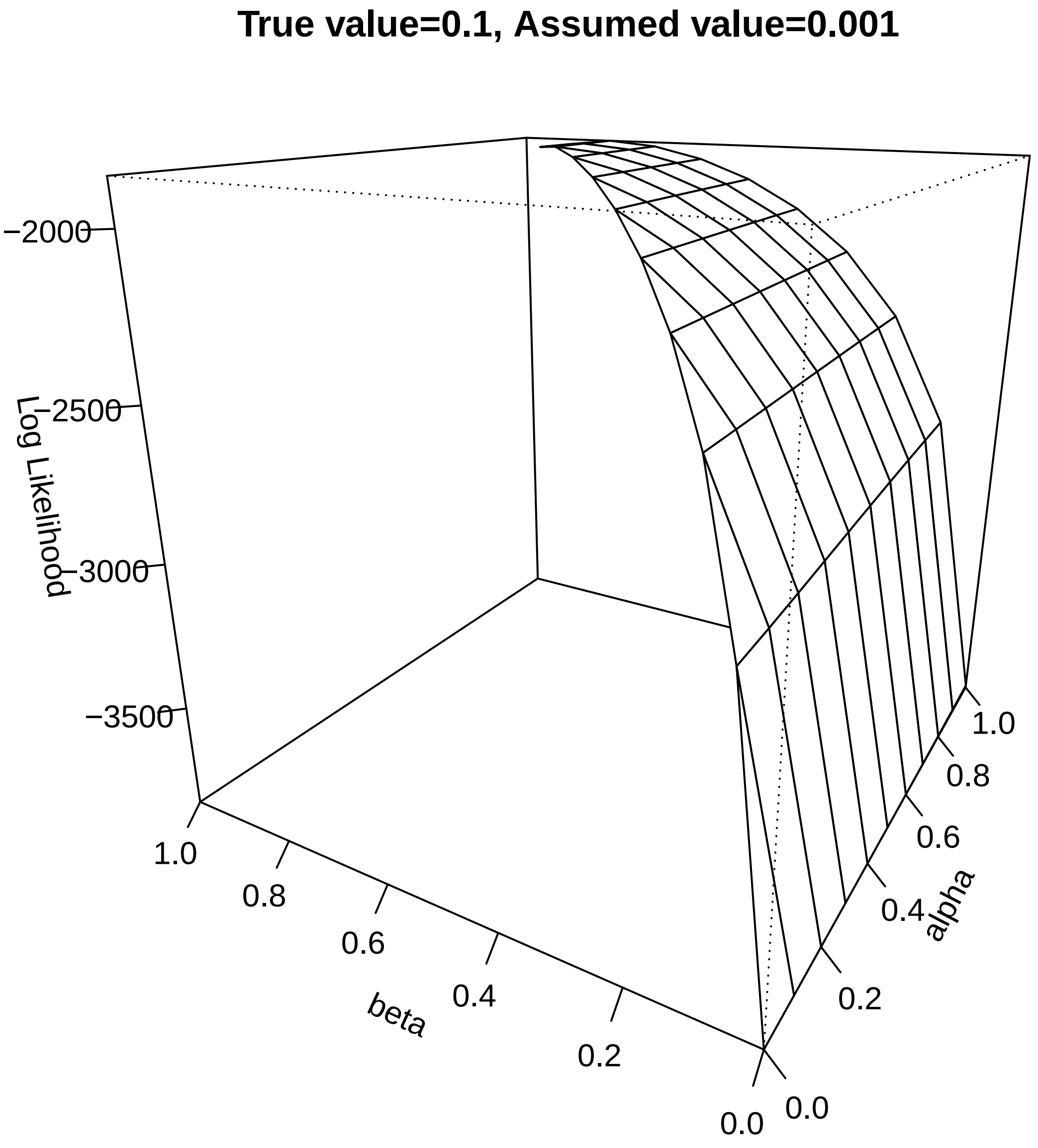
True value=0.1, Assumed value=0.001
Log Likelihood
−2000
−2500
−3000
−3500
beta
1.0
0.8
0.6
0.4
0.2
0.0
alpha
0.0
0.2
0.4
0.6
0.8
1.0

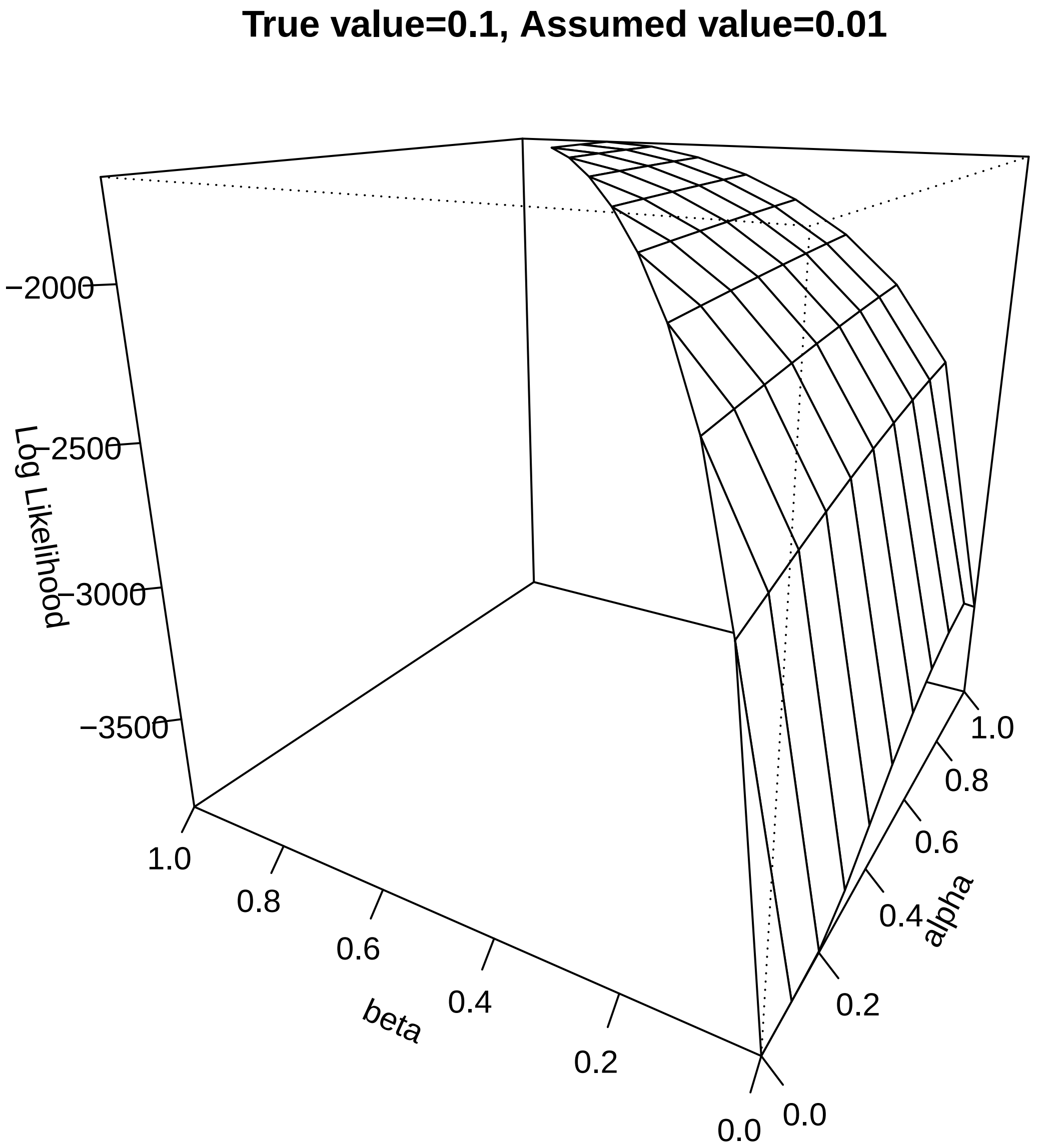

True value=0.1, Assumed value=0.01
Log Likelihood
−2000
−2500
−3000
−3500
beta
1.0
0.8
0.6
0.4
0.2
0.0
alpha
0.0
0.2
0.4
0.6
0.8
1.0

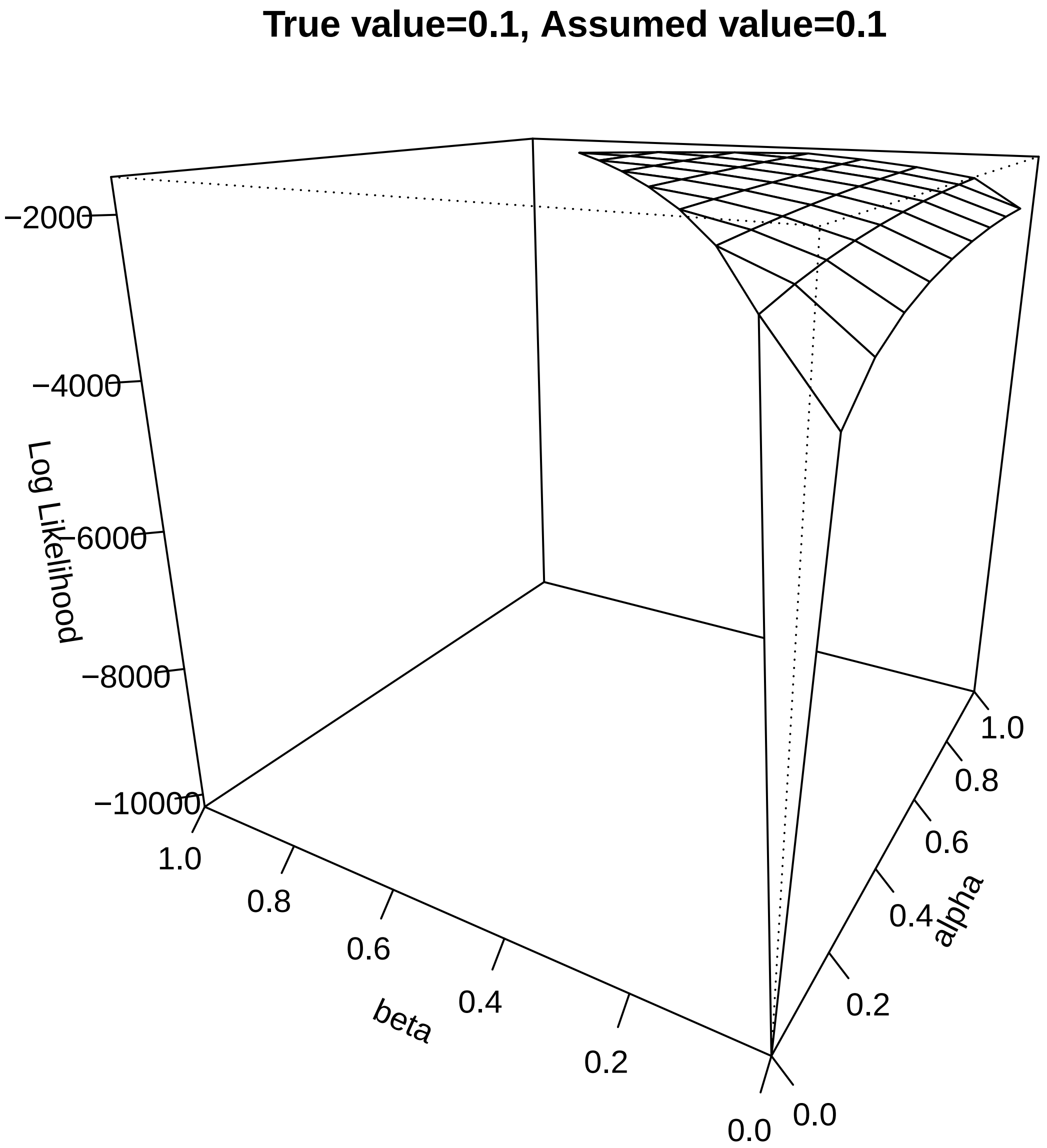
True value=0.1, Assumed value=0.1
Log Likelihood
−2000
−4000
−6000
−8000
−10000
beta
1.0
0.8
0.6
0.4
0.2
0.0
alpha
0.0
0.2
0.4
0.6
0.8
1.0

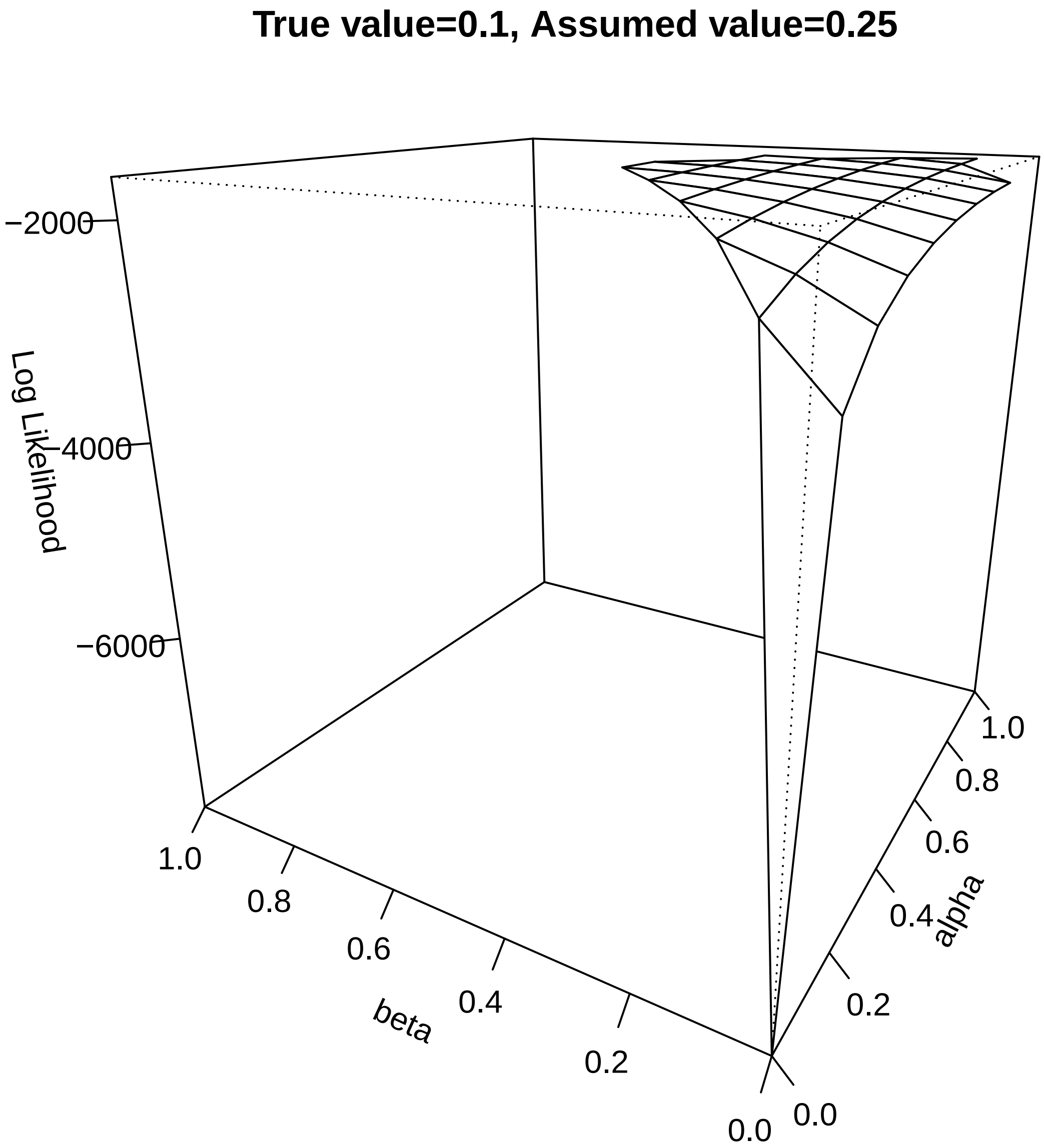

True value=0.1, Assumed value=0.25
Log Likelihood
−2000
−4000
−6000
beta
1.0
0.8
0.6
0.4
0.2
0.0
alpha
0.0
0.2
0.4
0.6
0.8
1.0

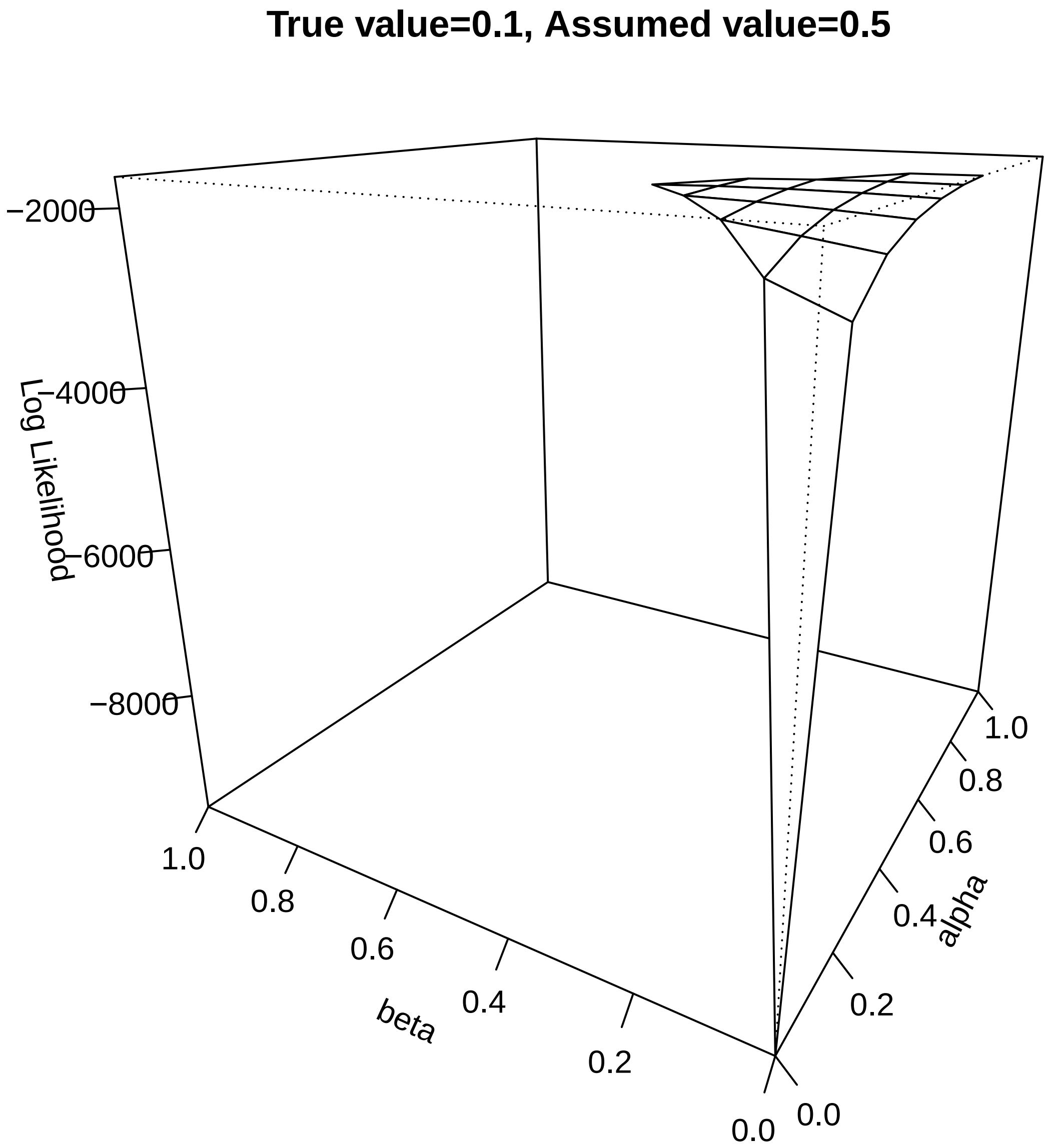
True value=0.1, Assumed value=0.5
Log Likelihood
−2000
−4000
−6000
−8000
beta
1.0
0.8
0.6
0.4
0.2
0.0
alpha
0.0
0.2
0.4
0.6
0.8
1.0

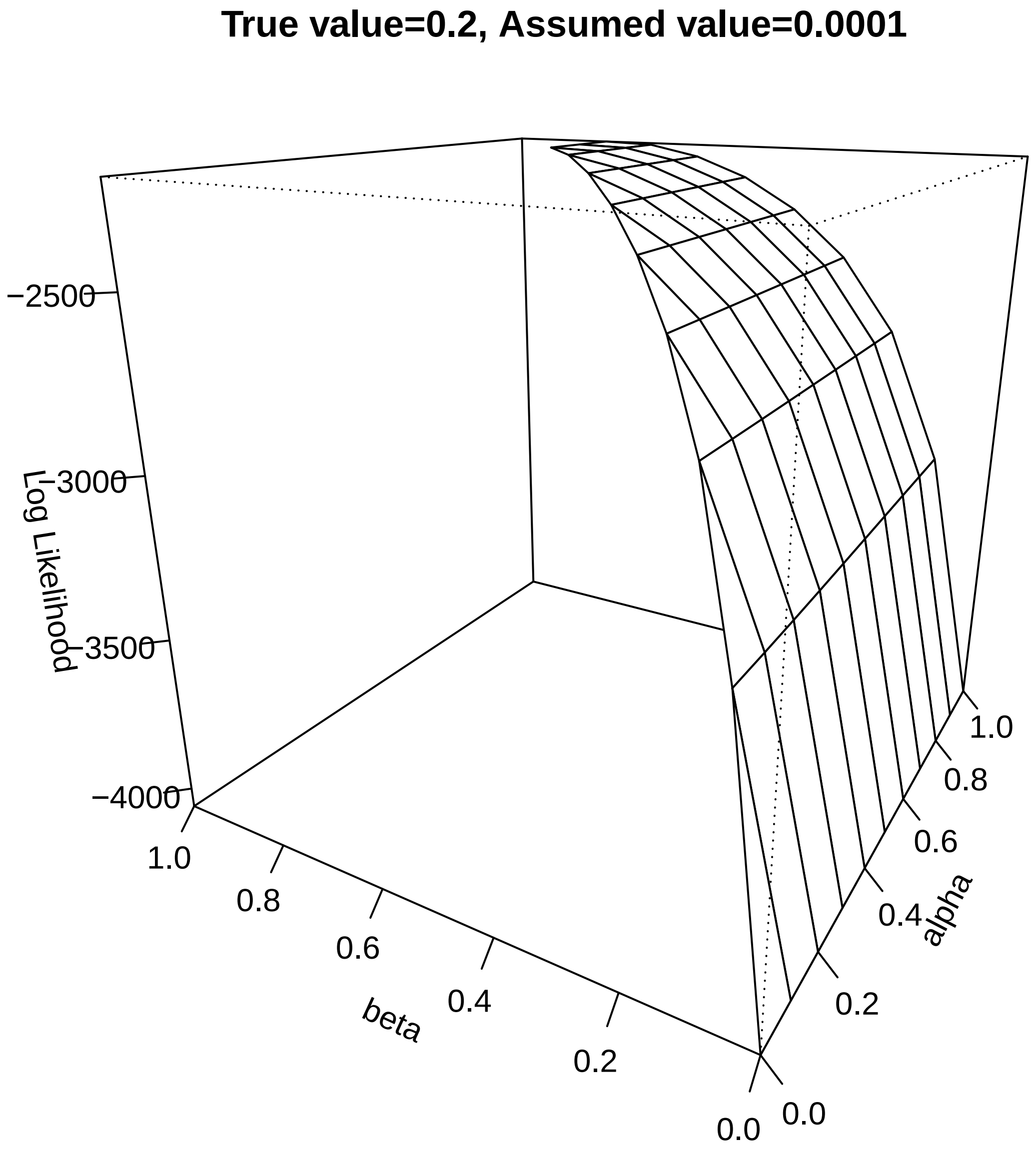
True value=0.2, Assumed value=0.0001
−2500
−3000
−3500
−4000
Log Likelihood
1.0
0.8
0.6
0.4
0.2
0.0
beta
0.0
0.2
0.4
0.6
0.8
1.0
alpha

True value=0.2, Assumed value=0.001

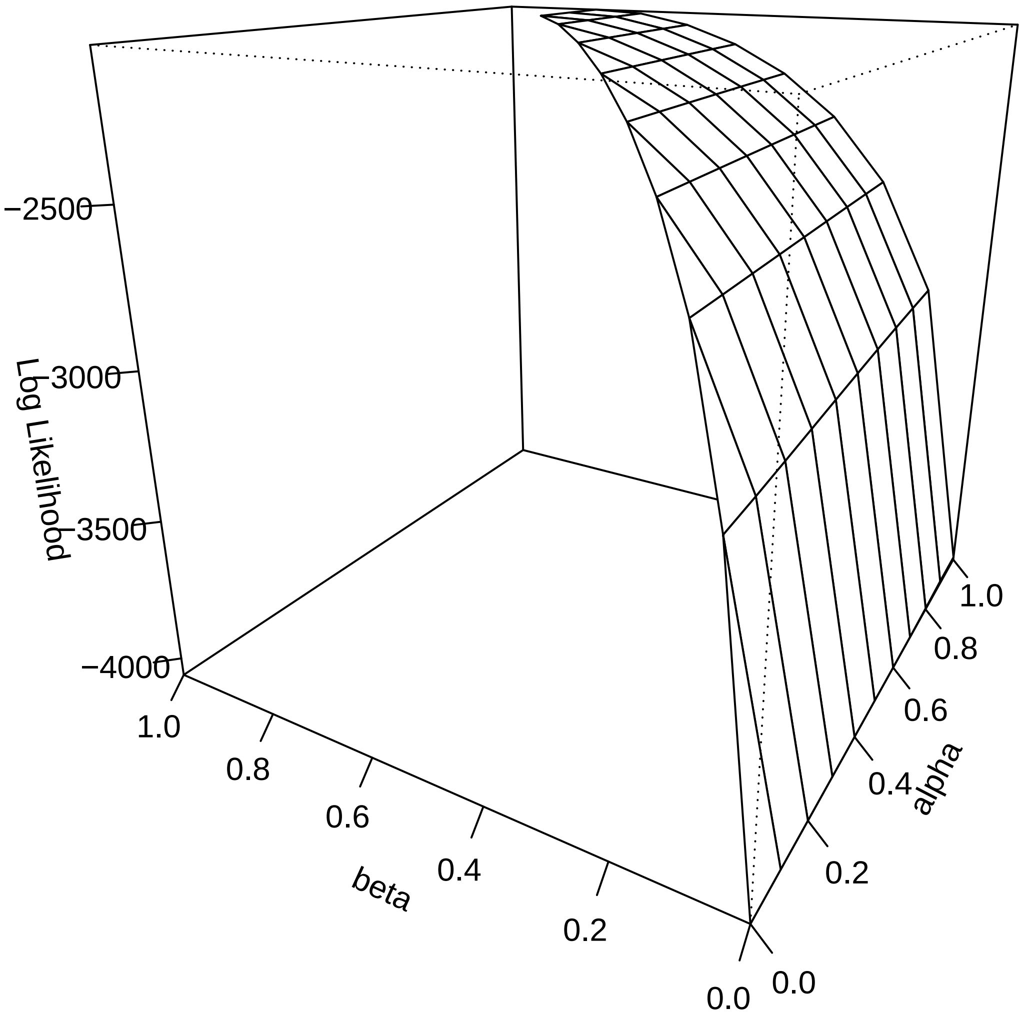

True value=0.2, Assumed value=0.01

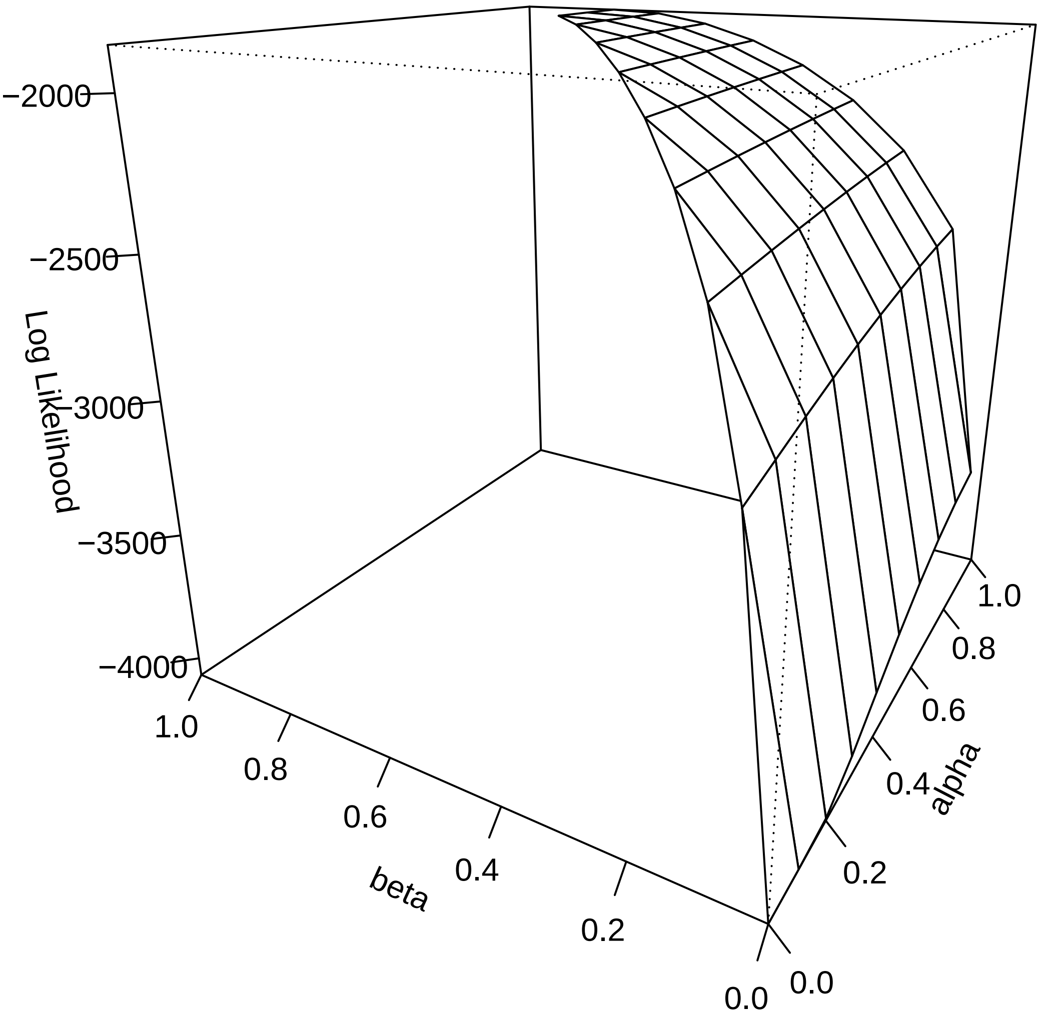

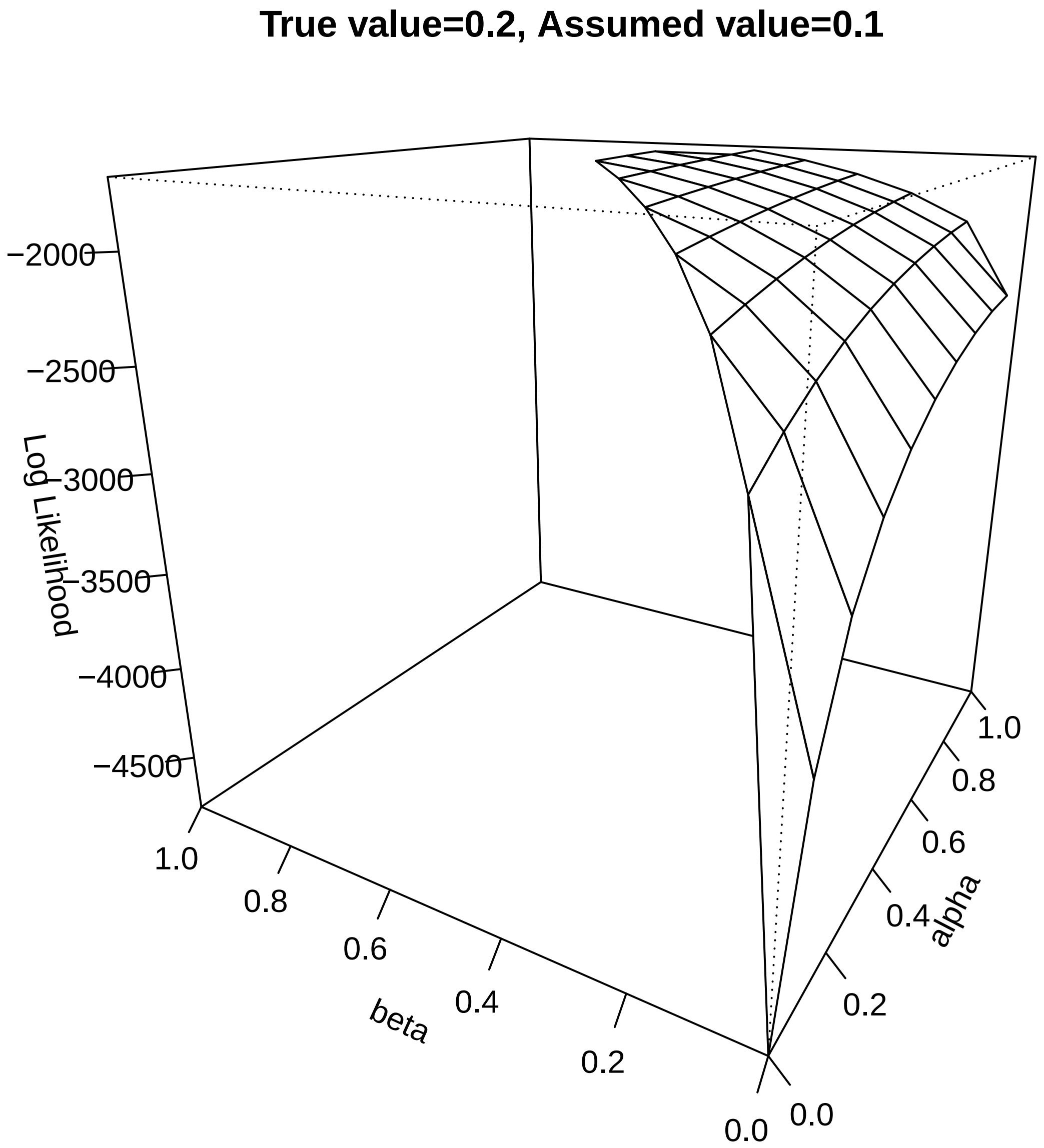
True value=0.2, Assumed value=0.1
−2000
−2500
−3000
−3500
−4000
−4500
Log Likelihood
1.0
0.8
0.6
0.4
0.2
0.0
beta
0.0
0.2
0.4
0.6
0.8
1.0
alpha

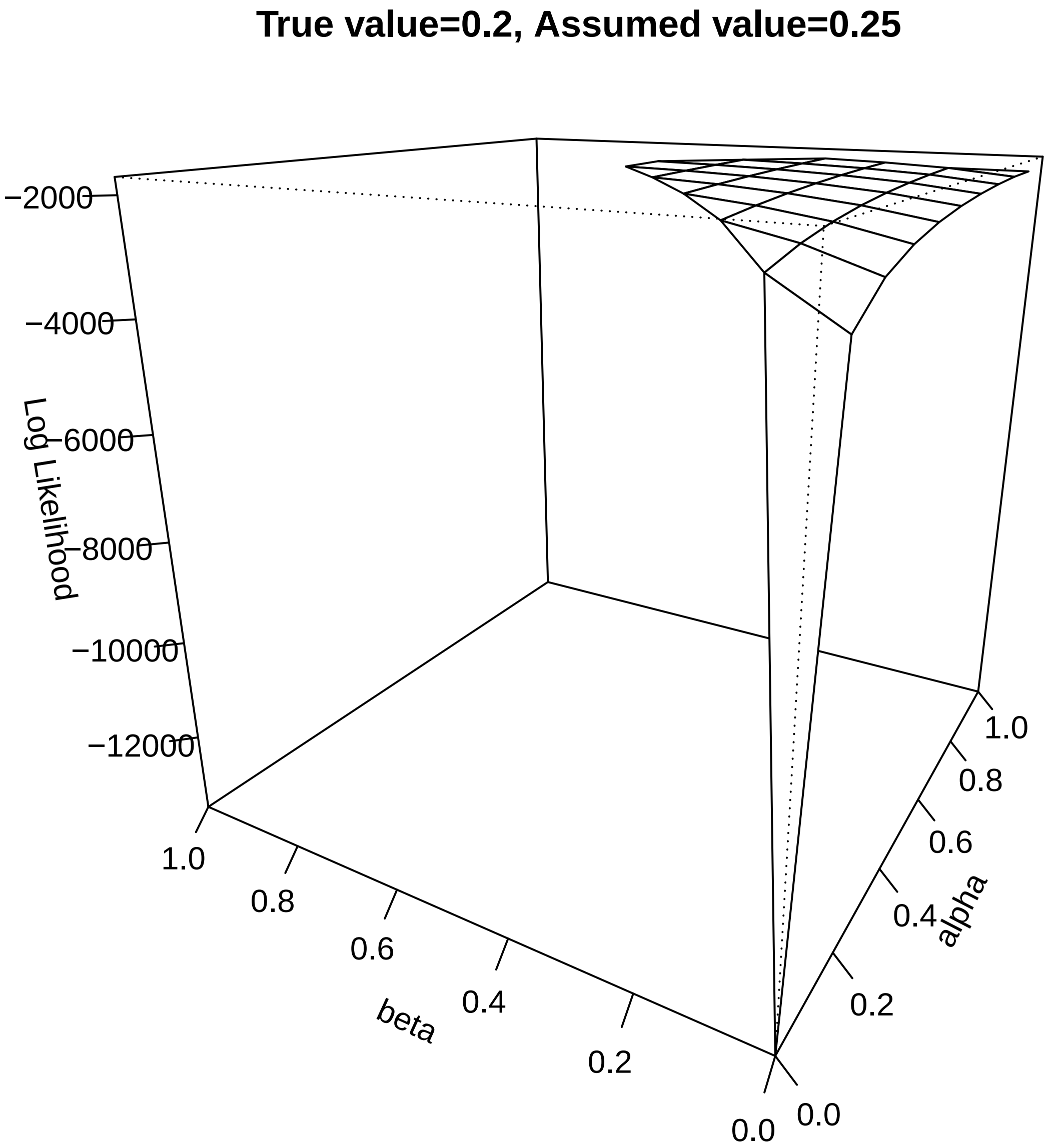
True value=0.2, Assumed value=0.25
Log Likelihood
−2000
−4000
−6000
−8000
−10000
−12000
beta
1.0
0.8
0.6
0.4
0.2
0.0
alpha
0.0
0.2
0.4
0.6
0.8
1.0

True value=0.2, Assumed value=0.5

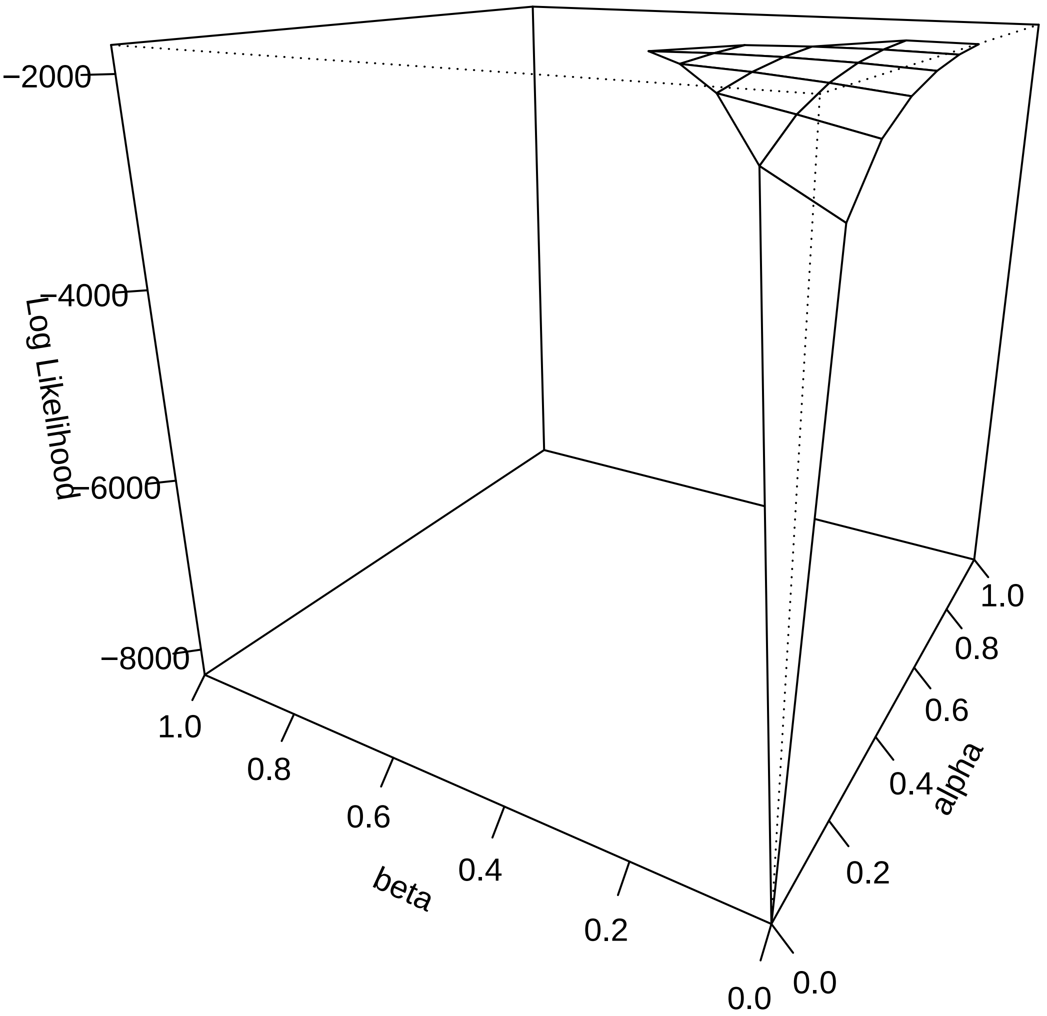

Supplement: Additional file 2 — Figure S1. Perspective plots of log likelihood surface for the simulation study of the penetrance model, 0.950, 0.700, and 0.000. The log likelihood surfaces are plotted for 30 cases of disease allele frequencies of the penetrance model, 0.950, 0.700, and 0.000. Fixing γ at its estimate evaluated under the constraint 0 ≤ γ ≤ 0.01, each log likelihood surface is drawn on a limited region α ≥ β. [file 1756-0500-5-465-S2.pdf]
