# Supplementary material for: MLEP: an R package for exploring the maximum likelihood estimates of penetrance parameters
Source: BMC Res Notes. 2012 Aug 28;5:465. doi: 10.1186/1756-0500-5-465 (PMC3537736; doi:10.1186/1756-0500-5-465)
Supplement: Additional file 3 — Table S1. Summary of maximum likelihood estimates of penetrance parameters for the simulation study of the penetrance model, 0.990, 0.900, and 0.000. The pedigree structure, number of marker alleles, marker allele frequencies, and recombination fractions are identical to those used in the simulation for the penetrance model, 0.950, 0.700, and 0.000, while penetrance parameters are assumed to be 0.990, 0.900, and 0.000. Six pedigree datasets with 50 pedigrees are generated, assuming disease allele frequencies to be 0.0001, 0.001, 0.01, 0.1, and 0.2. For each datasets, the likelihood polynomial is evaluated, assuming the frequency to be 0.0001, 0.001, 0.01, 0.1, 0.25, and 0.5, and the maximum likelihood estimates are evaluated under the constraint 0 ≤ γ ≤ 0.01. [file 1756-0500-5-465-S3.pdf]

Supplementary Table S1 : Summary of maximum likelihood estimates of penetrance parameters for the simulation study of the penetrance model, 0.990, 0.900, and 0.000.

True value	0.0001					
Assumed value	0.0001	0.001	0.01	0.1	0.25	0.5
MLE	(0.907,0.884,0.001)	(0.956,0.883,0.001)	(0.982,0.880,0.001)	(0.763,0.734,0.000)	(0.613,0.501,0.000)	(0.647,0.001,0.000)
Bias	(0.064,0.089,0.001)	(0.063,0.088,0.001)	(0.070,0.090,0.001)	(0.141,0.134,0.001)	(0.208,0.461,0.001)	(0.339,0.877,0.000)
MSE	(0.008,0.011,0.000)	(0.008,0.011,0.000)	(0.011,0.012,0.000)	(0.034,0.028,0.000)	(0.071,0.267,0.000)	(0.129,0.771,0.000)

True value	0.001					
Assumed value	0.0001	0.001	0.01	0.1	0.25	0.5
MLE	(0.980,0.884,0.001)	(0.986,0.884,0.001)	(0.987,0.881,0.001)	(0.749,0.744,0.000)	(0.831,0.274,0.000)	(0.664,0.000,0.000)
Bias	(0.064,0.089,0.001)	(0.062,0.088,0.002)	(0.069,0.090,0.001)	(0.137,0.135,0.001)	(0.205,0.461,0.001)	(0.337,0.878,0.000)
MSE	(0.008,0.011,0.000)	(0.008,0.011,0.000)	(0.010,0.012,0.000)	(0.034,0.028,0.000)	(0.069,0.268,0.000)	(0.128,0.772,0.000)

True value	0.01					
Assumed value	0.0001	0.001	0.01	0.1	0.25	0.5
MLE	(0.998,0.884,0.001)	(0.997,0.884,0.001)	(0.996,0.881,0.001)	(0.757,0.755,0.000)	(0.921,0.304,0.000)	(0.644,0.014,0.000)
Bias	(0.062,0.088,0.002)	(0.060,0.087,0.002)	(0.068,0.089,0.001)	(0.142,0.133,0.001)	(0.216,0.453,0.001)	(0.331,0.876,0.000)
MSE	(0.008,0.011,0.000)	(0.008,0.011,0.000)	(0.011,0.012,0.000)	(0.035,0.027,0.000)	(0.075,0.261,0.000)	(0.126,0.769,0.000)

True value	0.1					
Assumed value	0.0001	0.001	0.01	0.1	0.25	0.5
MLE	(1.000,0.883,0.010)	(1.000,0.890,0.008)	(0.999,0.885,0.002)	(0.785,0.734,0.000)	(0.580,0.513,0.000)	(0.658,0.007,0.000)
Bias	(0.061,0.088,0.003)	(0.053,0.088,0.003)	(0.061,0.090,0.003)	(0.143,0.125,0.002)	(0.211,0.427,0.001)	(0.319,0.864,0.001)
MSE	(0.007,0.011,0.000)	(0.006,0.011,0.000)	(0.009,0.011,0.000)	(0.033,0.024,0.000)	(0.071,0.233,0.000)	(0.121,0.750,0.000)

True value	0.2					
Assumed value	0.0001	0.001	0.01	0.1	0.25	0.5
MLE	(0.998,0.892,0.010)	(0.999,0.903,0.010)	(0.998,0.903,0.000)	(0.825,0.740,0.000)	(0.819,0.365,0.000)	(0.655,0.054,0.000)
Bias	(0.056,0.094,0.005)	(0.048,0.095,0.005)	(0.045,0.094,0.002)	(0.109,0.094,0.001)	(0.205,0.438,0.001)	(0.300,0.801,0.001)
MSE	(0.007,0.012,0.000)	(0.006,0.012,0.000)	(0.006,0.012,0.000)	(0.022,0.015,0.000)	(0.064,0.243,0.000)	(0.118,0.678,0.000)
