# Supplementary material for: MLEP: an R package for exploring the maximum likelihood estimates of penetrance parameters
Source: BMC Res Notes. 2012 Aug 28;5:465. doi: 10.1186/1756-0500-5-465 (PMC3537736; doi:10.1186/1756-0500-5-465)
Supplement: Additional file 4 — Table S2. Summary of LOD scores for the simulation study of the penetrance model, 0.990, 0.900, and 0.000. LOD scores are evaluated for 30 cases of disease allele frequencies for three penetrance models; true penetrance model (0.990, 0.900, and 0.000), dominant model (0.999, 0.999, and 0.000),and MLE model (the estimates are listed in Supplementary Table S1). [file 1756-0500-5-465-S4.pdf]

Supplementary Table S2 : Summary of LOD scores for the simulation study of the penetrance model, 0.990, 0.900, and 0.000.

Disease allele frequency		Model	Recombination fraction									
True value	Assumed value		0	0.05	0.1	0.15	0.2	0.25	0.3	0.35	0.4	0.45
0.0001	0.0001	True	5.576	5.226	4.812	4.349	3.842	3.295	2.707	2.081	1.419	0.724
		MLE	5.556	5.191	4.770	4.305	3.799	3.254	2.670	2.050	1.395	0.711
		Dominant	4.235	5.184	4.898	4.480	3.988	3.438	2.837	2.187	1.494	0.763
	0.001	True	5.574	5.225	4.810	4.347	3.841	3.294	2.706	2.080	1.418	0.724
		MLE	5.555	5.188	4.767	4.301	3.796	3.251	2.667	2.047	1.393	0.710
		Dominant	4.229	5.172	4.886	4.468	3.977	3.427	2.825	2.175	1.483	0.756
	0.01	True	5.559	5.210	4.796	4.333	3.827	3.280	2.693	2.068	1.408	0.717
		MLE	5.538	5.169	4.747	4.282	3.776	3.232	2.651	2.032	1.381	0.702
		Dominant	4.219	5.148	4.862	4.444	3.952	3.403	2.801	2.154	1.467	0.748
	0.1	True	5.427	5.080	4.668	4.209	3.707	3.166	2.587	1.973	1.328	0.665
		MLE	5.074	4.671	4.242	3.787	3.305	2.798	2.266	1.712	1.140	0.562
		Dominant	4.149	5.069	4.780	4.363	3.872	3.324	2.729	2.093	1.422	0.720
	0.25	True	5.213	4.871	4.466	4.014	3.522	2.992	2.427	1.833	1.218	0.598
		MLE	3.559	3.217	2.864	2.500	2.127	1.748	1.367	0.989	0.625	0.288
		Dominant	4.027	4.947	4.660	4.244	3.757	3.216	2.632	2.011	1.356	0.678
	0.5	True	4.791	4.466	4.077	3.644	3.174	2.671	2.141	1.591	1.036	0.494
		MLE	-0.951	-0.405	-0.152	-0.011	0.065	0.099	0.103	0.088	0.062	0.031
		Dominant	3.790	4.715	4.435	4.028	3.553	3.030	2.470	1.874	1.250	0.618
0.001	0.0001	True	5.576	5.227	4.812	4.349	3.843	3.295	2.708	2.081	1.419	0.724
		MLE	5.554	5.190	4.769	4.304	3.799	3.254	2.671	2.050	1.396	0.711
		Dominant	4.235	5.184	4.898	4.480	3.988	3.438	2.837	2.187	1.494	0.763
	0.001	True	5.575	5.225	4.811	4.348	3.841	3.294	2.706	2.080	1.418	0.724
		MLE	5.555	5.190	4.769	4.303	3.797	3.252	2.669	2.049	1.394	0.710
		Dominant	4.229	5.172	4.886	4.468	3.977	3.427	2.825	2.175	1.483	0.756
	0.01	True	5.560	5.210	4.796	4.333	3.827	3.280	2.694	2.068	1.408	0.717
		MLE	5.539	5.171	4.749	4.284	3.778	3.234	2.652	2.034	1.382	0.702
		Dominant	4.219	5.148	4.862	4.444	3.952	3.403	2.801	2.154	1.467	0.748
	0.1	True	5.427	5.080	4.669	4.209	3.708	3.166	2.587	1.973	1.328	0.665
		MLE	5.104	4.701	4.270	3.814	3.331	2.821	2.286	1.727	1.151	0.568
		Dominant	4.149	5.069	4.780	4.363	3.872	3.324	2.729	2.093	1.422	0.720
	0.25	True	5.213	4.872	4.466	4.014	3.522	2.992	2.427	1.833	1.218	0.598
		MLE	2.372	2.121	1.863	1.603	1.343	1.087	0.837	0.598	0.376	0.174
		Dominant	4.027	4.947	4.660	4.244	3.757	3.216	2.632	2.011	1.356	0.678
	0.5	True	4.791	4.466	4.077	3.644	3.174	2.671	2.141	1.591	1.036	0.494
		MLE	-1.233	-0.475	-0.186	-0.027	0.059	0.097	0.104	0.090	0.064	0.032
		Dominant	3.790	4.715	4.435	4.028	3.553	3.030	2.470	1.874	1.250	0.618
0.01	0.0001	True	5.387	5.086	4.684	4.233	3.739	3.204	2.631	2.020	1.375	0.701
		MLE	5.404	5.052	4.642	4.188	3.695	3.163	2.594	1.990	1.352	0.688
		Dominant	4.052	5.043	4.770	4.365	3.885	3.348	2.760	2.126	1.450	0.739
	0.001	True	5.406	5.085	4.683	4.231	3.737	3.203	2.630	2.019	1.374	0.700
		MLE	5.406	5.053	4.643	4.189	3.694	3.162	2.593	1.988	1.351	0.687
		Dominant	4.065	5.032	4.759	4.353	3.873	3.336	2.749	2.115	1.439	0.732
	0.01	True	5.411	5.074	4.670	4.218	3.724	3.190	2.618	2.008	1.365	0.693
		MLE	5.395	5.037	4.625	4.170	3.676	3.145	2.577	1.974	1.339	0.679
		Dominant	4.075	5.012	4.737	4.330	3.850	3.313	2.726	2.093	1.423	0.725
	0.1	True	5.299	4.957	4.553	4.102	3.611	3.082	2.516	1.917	1.289	0.644
		MLE	5.007	4.611	4.188	3.739	3.265	2.764	2.239	1.691	1.126	0.556
		Dominant	4.026	4.946	4.666	4.257	3.776	3.240	2.658	2.037	1.382	0.699
	0.25	True	5.095	4.758	4.360	3.916	3.434	2.915	2.364	1.784	1.184	0.581
		MLE	2.474	2.221	1.960	1.693	1.424	1.157	0.894	0.641	0.404	0.188
		Dominant	3.913	4.834	4.554	4.147	3.669	3.139	2.568	1.961	1.322	0.661
	0.5	True	4.682	4.362	3.981	3.557	3.098	2.607	2.089	1.553	1.011	0.483
		MLE	-0.301	-0.084	0.042	0.112	0.144	0.148	0.134	0.106	0.071	0.035

Supplementary Table S2 : Summary of LOD scores for the simulation study of the penetrance model, 0.990, 0.900, and 0.000.

Disease allele frequency		Model	Recombination fraction									
True value	Assumed value		0	0.05	0.1	0.15	0.2	0.25	0.3	0.35	0.4	0.45
		Dominant	3.688	4.615	4.342	3.945	3.479	2.967	2.419	1.836	1.226	0.606
0.1	0.0001	True	5.519	5.261	4.843	4.371	3.853	3.293	2.693	2.057	1.391	0.703
		MLE	5.383	5.025	4.606	4.145	3.646	3.108	2.535	1.933	1.310	0.666
		Dominant	4.214	5.232	4.933	4.500	3.991	3.424	2.806	2.145	1.452	0.735
	0.001	True	5.557	5.260	4.842	4.369	3.851	3.291	2.692	2.056	1.390	0.702
		MLE	5.489	5.130	4.705	4.234	3.722	3.174	2.589	1.973	1.330	0.671
		Dominant	4.248	5.222	4.922	4.488	3.980	3.413	2.795	2.134	1.442	0.729
	0.01	True	5.582	5.250	4.830	4.357	3.839	3.279	2.680	2.045	1.380	0.696
		MLE	5.554	5.195	4.768	4.293	3.777	3.221	2.629	2.003	1.350	0.679
		Dominant	4.277	5.203	4.900	4.466	3.957	3.389	2.772	2.114	1.428	0.722
	0.1	True	5.488	5.139	4.716	4.244	3.728	3.173	2.581	1.956	1.308	0.649
		MLE	5.098	4.694	4.259	3.796	3.307	2.791	2.252	1.693	1.122	0.550
		Dominant	4.247	5.143	4.832	4.394	3.884	3.318	2.707	2.061	1.388	0.696
	0.25	True	5.286	4.942	4.524	4.058	3.551	3.007	2.430	1.825	1.204	0.585
		MLE	3.614	3.267	2.906	2.533	2.151	1.764	1.376	0.994	0.626	0.288
		Dominant	4.139	5.033	4.721	4.284	3.777	3.219	2.620	1.986	1.327	0.656
	0.5	True	4.864	4.539	4.139	3.694	3.211	2.695	2.152	1.591	1.027	0.483
		MLE	-0.651	-0.298	-0.100	0.018	0.083	0.111	0.112	0.096	0.068	0.035
		Dominant	3.908	4.808	4.504	4.077	3.585	3.046	2.467	1.854	1.224	0.596
0.2	0.0001	True	5.220	5.021	4.633	4.188	3.697	3.165	2.593	1.985	1.345	0.681
		MLE	5.040	4.757	4.390	3.970	3.507	3.007	2.468	1.893	1.286	0.653
		Dominant	3.885	4.977	4.703	4.296	3.815	3.277	2.693	2.069	1.409	0.716
	0.001	True	5.279	5.030	4.634	4.188	3.697	3.164	2.593	1.985	1.345	0.681
		MLE	5.230	4.921	4.532	4.092	3.610	3.088	2.529	1.935	1.311	0.663
		Dominant	3.948	4.977	4.702	4.295	3.814	3.277	2.692	2.069	1.408	0.716
	0.01	True	5.329	5.039	4.634	4.182	3.689	3.157	2.586	1.978	1.339	0.677
		MLE	5.329	5.042	4.639	4.187	3.695	3.162	2.590	1.981	1.342	0.679
		Dominant	4.014	4.975	4.698	4.290	3.809	3.272	2.688	2.065	1.405	0.713
	0.1	True	5.293	4.968	4.562	4.108	3.614	3.082	2.515	1.915	1.287	0.643
		MLE	4.980	4.590	4.168	3.720	3.245	2.746	2.223	1.679	1.119	0.553
		Dominant	4.035	4.937	4.647	4.238	3.758	3.223	2.642	2.023	1.370	0.690
	0.25	True	5.149	4.818	4.414	3.964	3.476	2.952	2.395	1.809	1.202	0.590
		MLE	2.907	2.609	2.305	1.996	1.684	1.373	1.065	0.767	0.484	0.225
		Dominant	3.964	4.846	4.553	4.144	3.667	3.137	2.562	1.953	1.313	0.654
	0.5	True	4.818	4.494	4.101	3.665	3.194	2.690	2.159	1.606	1.045	0.498
		MLE	0.417	0.433	0.420	0.389	0.345	0.293	0.234	0.173	0.112	0.053
		Dominant	3.784	4.654	4.367	3.964	3.495	2.977	2.418	1.830	1.219	0.599
